# Cross-Kingdom Interaction of miRNAs and Gut Microbiota with Non-Invasive Diagnostic and Therapeutic Implications in Colorectal Cancer

**DOI:** 10.3390/ijms241310520

**Published:** 2023-06-23

**Authors:** Ondrej Pös, Jakub Styk, Gergely Buglyó, Michal Zeman, Lydia Lukyova, Kamila Bernatova, Evelina Hrckova Turnova, Tomas Rendek, Ádám Csók, Vanda Repiska, Bálint Nagy, Tomas Szemes

**Affiliations:** 1 Comenius University Science Park, 841 04 Bratislava, Slovakia; jakub.styk@uniba.sk (J.S.); michal.zeman65@uniba.sk (M.Z.); evelina.turnova@gmail.com (E.H.T.); nagy.balint@med.unideb.hu (B.N.); tomas.szemes@geneton.sk (T.S.); 2 Geneton Ltd., 841 04 Bratislava, Slovakia; 3 Institute of Medical Biology, Genetics and Clinical Genetics, Faculty of Medicine, Comenius University, 811 08 Bratislava, Slovakia; rendektomas@gmail.com (T.R.); vanda.repiska@fmed.uniba.sk (V.R.); 4 Department of Human Genetics, Faculty of Medicine, University of Debrecen, 4032 Debrecen, Hungary; gbuglyo80@gmail.com (G.B.); csok.adam@med.unideb.hu (Á.C.); 5 Department of Molecular Biology, Faculty of Natural Sciences, Comenius University, 842 05 Bratislava, Slovakia; lukyova6@uniba.sk (L.L.); kamila.bernatova@gmail.com (K.B.); 6 Slovgen Ltd., 841 04 Bratislava, Slovakia; 7 Medirex Group Academy, n.p.o., 949 05 Nitra, Slovakia

**Keywords:** colorectal cancer, biomarkers, microRNAs, gut microbiota, dysbiosis

## Abstract

Colorectal cancer (CRC) has one of the highest incidences among all types of malignant diseases, affecting millions of people worldwide. It shows slow progression, making it preventable. However, this is not the case due to shortcomings in its diagnostic and management procedure and a lack of effective non-invasive biomarkers for screening. Here, we discuss CRC-associated microRNAs (miRNAs) and gut microbial species with potential as CRC diagnostic and therapy biomarkers. We provide rich evidence of cross-kingdom miRNA-mediated interactions between the host and gut microbiome. miRNAs have emerged with the ability to shape the composition and dynamics of gut microbiota. Intestinal microbes can uptake miRNAs, which in turn influence microbial growth and provide the ability to regulate the abundance of various microbial species. In the context of CRC, targeting miRNAs could aid in manipulating the balance of the microbiota. Our findings suggest the need for correlation analysis between the composition of the gut microbiome and the miRNA expression profile.

## 1. Introduction

Colorectal cancer (CRC) shows slow progression since it takes several years to develop from a benign adenoma and progress into a malignant adenocarcinoma [1]. This lengthy process makes it possible to use screening programs and prevent the disease in the early stage when the treatment is more effective and leads to better health outcomes. Unfortunately, there are many shortcomings with the current screening approaches, such as colonoscopy, which is invasive, time-consuming, and unpleasant, but it is still the gold standard procedure [2]. Currently, the implementation of population-based screens for CRC, such as colonoscopy or flexible sigmoidoscopy, is associated with low compliance rates, and non-invasive immunochemical fecal occult blood tests (iFOBTs) have become globally acknowledged screening methods for CRC detection [3,4]. However, their sensitivity in the detection of early premalignant colorectal adenomas (CRAs) or early stage CRC is poor, and it has been reported that almost 40% of CRC cases could be undetected [5,6,7].

Early CRC detection enhances the overall survival, prognosis, and curability of CRC patients [8,9,10]. Thus, introducing non-invasive and sensitive tests for the early detection of CRC lesions is fundamental to reducing the number of unnecessary colonoscopies and preventable deaths. Multi-panel biomarkers (e.g., genomic, transcriptomic, proteomic, inflammatory, and microbiome) combined with non-invasive sample collection from peripheral blood or stool are promising for early CRC detection. Due to its attractive characteristics (e.g., abundance and stability), microRNA (miRNA) seems to be a reasonable liquid-biopsy-based biomarker of malignant processes [11,12]. On the other hand, the gut microbiome composition has also been suggested as a potential marker of tumorigenesis [13]. Since the microbiota may affect important physiological and pathophysiological processes and, at the same time, interact with host miRNAs, the evaluation of both multi-omics biomarkers could provide a novel approach for early cancer diagnosis or patient follow-up. Although bacteria-induced cancer progression is still not fully understood, it is known that some species are able to facilitate tumorigenesis in an miRNA-dependent manner. We summarize the CRC-associated crosslinks between miRNA and the host-microbiome composition to highlight biomarkers that could provide potential biomedical applications in oncology.

## 2. microRNAs in Body Fluids

miRNAs are short (approximately 22 nt long) non-coding RNA (ncRNA) molecules of endogenous origin [14]. By binding to various genomic regions, they regulate the expression of genes involved in not only normal healthy conditions but also in the pathogenesis of severe diseases, including cancer [15]. Since miRNAs may be encapsulated in exosomes or associated with proteins (e.g., AGO2 or NPM1), they are well preserved and resistant to RNase-mediated degradation in body fluids [16]. Moreover, they were shown to be stable during long-term storage and even after repeated freezing and thawing [17]. Such biological characteristics predispose miRNA molecules to be ideal biomarkers for non-invasive liquid-biopsy-based cancer assessment strategies.

**Figure 1:**
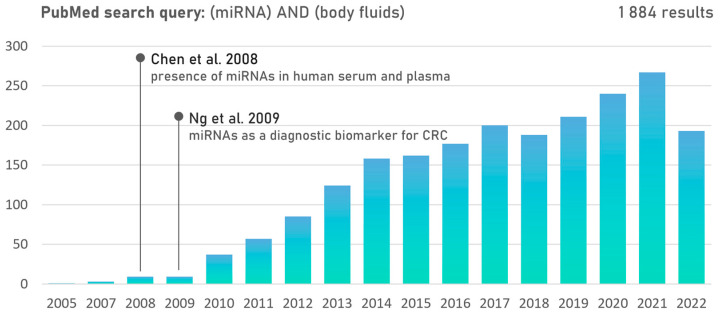
PubMed publication records searching for the terms (miRNA) AND (body fluids) suggest emerging research interest in cell-free miRNAs since 2009 [18,19].

The study by Chen et al. was the first study to demonstrate the presence of miRNAs in human serum and plasma [18]. Moreover, specific expression patterns of serum miRNAs for various diseases, including CRC, have been identified. Ng et al. made the first systematic and comprehensive analysis of miRNA applicability and found miR-17-3p and miR-92 to be significantly elevated in CRC patients while recommending plasma miR-92 as a novel diagnostic biomarker for CRC [19]. Motivated by potential biomedical applications, such conclusions unleashed extensive research on miRNAs in body fluids (Figure 1).

### 2.1. Role of miRNAs in CRC Development

The aberrant expression of miRNAs was shown to play a role in the initiation and progression of CRC. The functions of dysregulated miRNAs appear to be context-specific, having a dual role in tumor development. A comprehensive list of both tumor-suppressive and tumor-promoting miRNAs associated with CRC development was depicted in the review by Ding et al. [20]. Several miRNAs act as oncogenes by inhibiting the expression of tumor-suppressor genes; thus, they are known as “oncomiRs” [21,22]. One of the most widely studied oncomiRs is miR-21, which mediates colorectal tumorigenesis by regulating the mitogen-activated protein kinases (MAPK) pathway and WNT/β-Catenin signaling through targeting PTEN, PDCD, and DKK2 [23]. The expression levels of miR-21 allow the discrimination of patients with CRA and CRCs from healthy volunteers, suggesting it is a promising biomarker for colorectal neoplasia screening. In favor of liquid biopsy, miR-21 is released by cancer cells and was shown to be abundant in plasma and serum [24,25]. Another oncomiR example associated with CRC and CRA is miR-135b, whos detection in stool samples has been proposed as a non-invasive biomarker for CRC and CRA [26], while targeting miR-135b was suggested as a possible treatment strategy for reversing oxaliplatin resistance in CRC [27]. Although some oncomiRs (e.g., miR-21, miR-155, and miR-224) have been reported to be consistently upregulated in the circulation of cancer patients, the specificity of these miRNAs is still questionable as they have been connected to inflammation and showed elevated levels in inflammatory diseases [28,29].

On the other hand, miRNAs may also provide tumor-suppressive characteristics through downregulating oncogenes associated with proliferation, apoptosis, invasion, and migration. A well-studied representative is miR-34a, which suppresses CRC metastasis by targeting Notch1/Jagged1 and thus regulates their downstream molecules vimentin and fibronectin [30]. In accordance, tumor-derived exosomes encapsulating miR-34a were shown to promote apoptosis and inhibit the migration and tumor progression of CRC cells [31].

However, research on the role of some miRNAs in oncogenesis is contradictory. For example, RAC1-activation-promoting cellular invasion [32], as well as RAC1 inhibition suppressing cell proliferation, invasion, and epithelial-to-mesenchymal transition [33] via miR-142-3p, has been described. Another example is miR-155, which was demonstrated as a tumor suppressor in CRC by targeting CTHRC1 [34]. In contrast, Zhang et al. have shown that the upregulation of miR-155 could promote the migration and invasion of CRC cells through the regulation of claudin-1 expression [35], and Wan et al. indicated it as a potential contributor to the progression and growth of CRC by enhancing the Wnt/β-catenin pathway in an HMG-box-transcription-factor-1-associated mechanism [36].

The contradicting levels of miRNAs in different CRC studies may be partially explained by a dynamic relationship between miRNAs and competitive endogenous RNAs (miRNA sponges). The colon and rectum are highly dynamic environments that respond to various environmental factors and thus require a rapidly modulable nature, providing the ability to induce or negate the effect of mRNA translation. Such conditions predispose ncRNA to be an ideal molecule for enabling the miRNA-mediated mRNA degradation and competitive sequestration of miRNAs, leading to gene downregulation and upregulation, respectively [37].

### 2.2. Non-Invasive miRNA Biomarkers for Early CRC Diagnosis

Tissue-based studies have found several differentially expressed miRNAs involved in the cascade of colorectal carcinogenesis that may serve as diagnostic biomarkers for CRC patients [38]. Those miRNAs circulating in body fluids provide promise as non-invasive biomarkers accessible through liquid biopsy (Table S1). Several serum miRNAs were suggested for reliable differentiation between CRC patients and control groups, including miR-17, miR-19a, miR-20a, and miR-223 [39]; a panel of miR-31, miR-141, miR-224-3p, miR-576-5p, and miR-4669 [40]; or a panel of exosomal miR-19a, miR-20a, miR-143, miR-145, miR-150, and let-7a [41]. For example, a promising diagnostic panel combining miRNAs (downregulated miR-144-3p, miR-425-5p, miR-1260 and upregulated miR-19a, miR-19b, miR-15b, miR-29a, miR-335, and miR-18a) detectable in plasma or serum samples demonstrated a sensitivity and specificity over 90% [42]. Thus, a multimarker evaluation seems beneficial for increasing the sensitivity and/or specificity of screening tests.

Another complicating factor in the search for biomarkers is the observation that the expression levels of miRNAs exhibit dynamics through the stages of CRC. Systematic increases in the levels of miR-21, miR-31, miR-20a, and miR-135b were observed in plasma during various stages of CRC [43]. These changes were particularly noticeable during the transition from an adenoma to a malignant tumor and the onset of lymph node metastasis, ultimately leading to distant metastasis, as evidenced by high serum levels of miR-200c in stage IV CRC patients [44]. Notably, similar patterns of significant changes in miRNA expression were observed in the alterations of the microbiome in CRC patients [45,46]. However, the current understanding lacks mechanistic studies to confirm or disprove the causality between these miRNA changes and microbiome alterations in the different stages of CRC.

Fecal sampling is also a non-invasive alternative for miRNA detection. Several miRNAs, including miR-223, miR-451 [47], miR-29a, miR-135b, and miR-224, are detectable in feces and may facilitate the non-invasive screening or diagnosis of CRC [48]. Fecal miR-106a was shown to be a useful marker to identify CRC patients with false-negative iFOBT results. Thus, miRNA tests combined with iFOBT may improve the sensitivity of CRC detection [49]. However, as gut bleeding is a clinically prevalent phenomenon of CRC, blood in stool may affect fecal miRNA levels to a varying extent and may substantially impact the interpretation of the clinical data. Considering the propensity of gut bleeding in CRC, the observed upregulation of some fecal miRNAs in CRC patients may be attributed predominantly to blood in their stool. Thus, the effect of blood in stool on the levels of fecal miRNA markers should be considered in the future design of clinical fecal miRNA profiling studies [47].

Promising studies have assessed miRNAs as potential biomarkers for several conditions [50]. Unfortunately, each scientific group used a different laboratory-specific pre-analytical setup for sample collection, total RNA extraction, cDNA synthesis, reverse transcription, and quantitative real-time polymerase chain reaction (qPCR) analysis. This lack of consensus in protocols does not allow for tracking and reproducing setups between labs [51,52]. Since the pre-analytical processes such as normalization and quantification could be different, the careful assay optimization and standardization of internationally accepted protocols are needed for a successful comparison of results produced by other laboratories [53,54,55,56].

## 3. Dynamics of Gut Microbiota Composition

The human microbiome is composed of a wide range of microorganisms, including viruses, fungi, archaea, and bacteria, with bacteria being the most numerous group [57,58,59]. The human body contains more than 100 trillion microbes [60,61], most of which (approx. 10^13^–10^14^ microorganisms) occur in the intestines and are composed of more than 1000 different bacterial species. Interactions between the host and the microbiome are dynamic and depend on several genetic and environmental factors, such as age, geographic location, ethnicity, and lifestyle (e.g., diet, alcohol or drug intake) [62,63,64,65,66]. Intestinal microorganisms are essential in human physiology and metabolism, immune system modulation, and the competitive exclusion of enteropathogenic bacteria [60,61]. When the balance between the microbiome and the host is disturbed, dysbiosis occurs, causing severe problems, including cancer development [67,68]. Mechanisms by which a dysbiotic microbiome may contribute to CRC include changes in the immune system regulation, chronic inflammation, or the modification of various food component metabolisms, which may lead to toxic metabolite or genotoxin production [69,70,71]. Considering the bacterial driver–passenger model in CRC development, bacteria are divided into directly procarcinogenic (drivers), causing changes in the tumor microenvironment, and indirectly procarcinogenic (passengers), which proliferate as opportunistic pathogens in the tumor-associated microenvironment. Interactions between microorganisms and the host may contribute to the activation of signaling pathways, leading to the development and subsequent progression of tumor growth [72].

### 3.1. Gut-Microbiota-Driven CRC Development and Progression

Although the composition of the intestinal microbiome is known to be individual and vary over time, the two most common phyla are Firmicutes and Bacteroidetes, which account for approximately 90% of all the bacteria present in healthy adults. Other members of a healthy microbiome in the colon include *Eubacterium*, *Bifidobacterium*, *Fusobacterium*, *Lactobacillus*, *Enterococcus*, *Streptococcus*, and Enterobacteriaceae [73,74,75]. Testing of stool and tumor samples from CRC patients has revealed differences in microbiome composition when compared to healthy controls. These differences were mostly in the taxons *Bacteroides fragilis*, *Streptococcus gallolyticus*, *Enterococcus faecalis*, *Escherichia coli*, *Fusobacterium nucleatum*, *Parvimonas*, *Peptostreptococcus*, *Porphyromonas*, and *Prevotella* (Table 1) [71,76,77,78,79,80]. The microbial composition also varies depending on the stage of the disease. For example, *F. nucleatum* or *Solobacterium moorei* strains are extensively represented in the early and metastatic stages of CRC, while *Atopobium parvulum* and *Actinomyces odontolyticus* strains are more prevalent in patients with CRA and intramucosal carcinomas [46,76]. Thus, the development of the CRC is not caused by one specific microorganism but involves a set of different bacteria whose harmful influence outweighs the influence of beneficial species. A reduced proportion of these beneficial bacteria, such as *Clostridium butyricum* or *Streptococcus thermophilus,* has been observed in patients with CRC [81,82,83]. The detection of significant microbiome changes in patients with CRC can serve not only as a biomarker for disease screening but also as a predictor of the response to treatment or as a way of determining the prognosis of individual patients [84]. However, a major barrier to analyzing the gut microbiome as a cancer biomarker is often non-compliance due to the undesirable nature of stool sample collection, which can be challenging for a patient [85].

Additionally, there is growing evidence that fungi play an important role in oncogenesis. Two large meta-studies recently showed that there are cancer-type-specific fungal communities even in colon cancers. Tumors and their adjacent tissue from the colon are dominated by *Candida*, *Malassezia*, and *Saccharomyces* genera, although in general, fungal abundances are lower than those of bacteria [86,87].

**Table 1:** Comparison of bacterial species identified from stool and tissue samples in patients with CRC and healthy individuals.

Bacterial Species	Presence in CRC Patients	Oncogenic Mechanism	Reference
*Bacteroides fragilis*	up	Wnt signaling activation, toxigenic	[88,89,90]
*Enterococcus faecalis*	up	pro-inflammatory signaling	[91,92,93]
*Escherichia coli*	up	Wnt signaling activation, genotoxicity	[94,95,96]
*Fusobacterium nucleatum*	up	Wnt signaling activation	[77,79,91,97,98]
*Solobacterium moorei*	up	unknown	[79,99]
*Streptococcus gallolyticus*	up	pro-inflammatory signaling	[100,101]

## 4. miRNAs in Host-Gut Microbiota Communications

**Figure 2:**
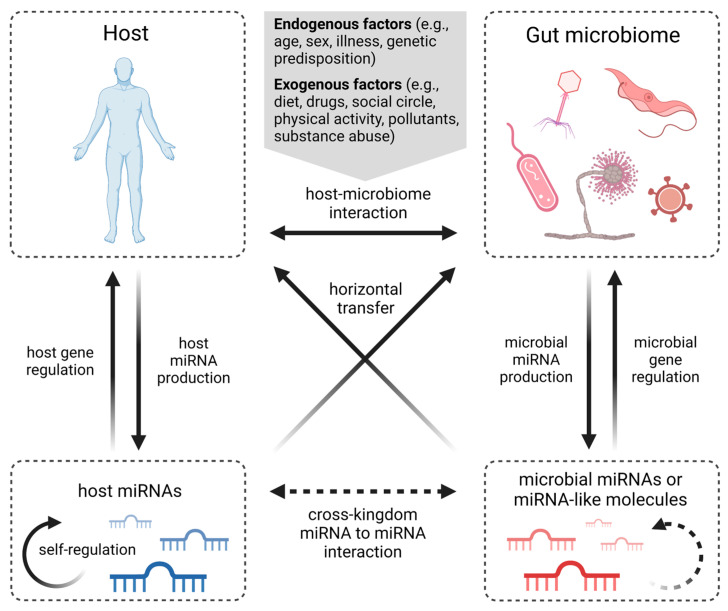
Known types of miRNA-mediated interactions between host and gut microbiota. Human-host–microbiome communication is affected by endogenous and exogenous factors. Both host and microorganisms produce miRNAs to regulate gene expression, while miRNA self-regulation has also been described. However, host-derived miRNAs have shown the ability to regulate microbiome gene expression, and microbial miRNAs and miRNA-like molecules can regulate gene expression of the host. Although self-regulation of microbial miRNAs, as well as interactions between host miRNAs and microbial miRNA molecules, can be hypothesized, these mechanisms have yet to be confirmed. Moreover, several cross-kingdom miRNA interactions have been described, but such communication in the gut microbiome is understudied (created with BioRender.com).

Host and gut microbiome interactions can be mediated by proteins, metabolites, and ncRNAs, e.g., miRNA, circular RNA, and long non-coding RNA (lncRNA) [102,103]. Recent evidence has revealed a bidirectional interaction between miRNA expression and the human gut microbiota composition [104] (Figure 2). Several cancer-related bacteria, including *F. nucleatum*, *E. coli*, *B. fragilis*, and *Faecalibacterium prausnitzii*, were shown to modulate miRNA levels in CRC cells, thus playing a critical role in the disturbance of intestinal homeostasis (Table 2). Commensal bacteria can produce extracellular vesicles carrying RNA molecules with a gene expression regulatory ability that can be delivered to host cells and regulate the expression of genes regulating the efficacy of cancer therapy [105]. On the other hand, specific host miRNAs can be taken up by bacteria, where they regulate microbial gene expression and subsequently may promote gut dysbiosis [106]. Although the mechanism is poorly understood, specific microbiome–miRNA interactions are able to mediate gastrointestinal cancer development or cancer therapy resistance, and thus bacterial-strain–miRNA correlations could represent a novel diagnostic or therapeutic strategy.

### 4.1. Microbiota–miRNA Interaction in Host Metabolism

**Figure 3:**
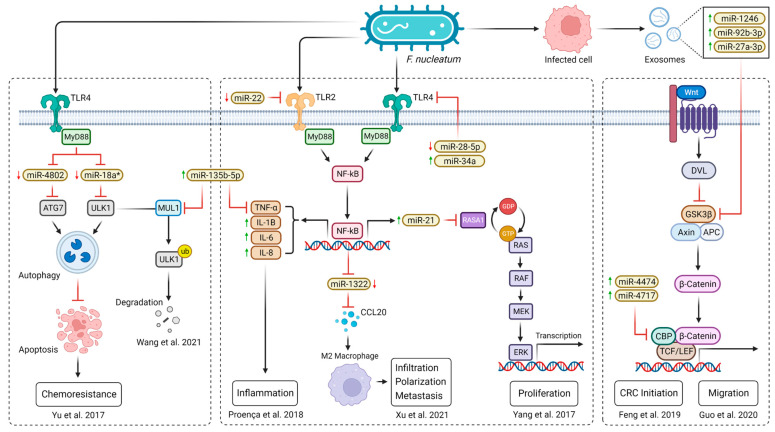
A scheme of *F. nucleatum*-mediated CRC development via miRNA interactions. However, not all the proposed miRNA interactions have yet been confirmed by functional studies [27,108,109,112,113,114,115]. Explanatory notes: red arrows (↓) represent decreased level; green arrows (↑) represent increased level; black arrows describe direct stimulatory modification or transcriptional activation of gene expression; red bunt-ended lines (T) describe direct inhibitory effect (created with BioRender.com).

A common denominator associated with CRC through miRNA interactions is the presence of *F. nucleatum*, an anaerobic oral commensal that is considered a pathogenetic factor contributing to multiple gastrointestinal disorders, including CRC [107]. Yu et al. proposed a pathway of the *F. nucleatum*-mediated activation of TLR4 and MyD88 signaling, causing the genomic loss of mir-18a* and mir-4802 (molecules targeting the autophagy-related proteins ULK1 and ATG7, respectively), resulting in autophagy activation and consequently promoting chemoresistance to oxaliplatin and 5-FU in CRC patients [108] (Figure 3). Another molecule promoting oxaliplatin resistance in CRC is miR-135b-5p, which was shown to be upregulated in CRA and CRC cells together with the overabundance of *F. nucleatum* in the tumor microenvironment [109]. Its direct downstream target, MUL1, acts as a ubiquitin ligase and thus could degrade ULK1, resulting in protective autophagy induction [27]. On the other hand, a negative correlation between miR-135b-5p and TNF-α with a potential immunomodulatory effect in CRC development was observed [109] and was supported by an miR-135b-5p inhibitory effect on lipopolysaccharide-induced TNFα production in human macrophages [110].

An increased expression of miR-21, miR-17-5P, and miR-155, the molecules that can affect the metabolism of tumor cells, was also associated with the presence of *F. nucleatum* in CRC biopsy samples [111]. The upregulation of miR-21 takes place in a promoter-dependent manner by NF-κB stimulation via *F. nucleatum*-activated TLR4/MyD88 cascade. Subsequently, miR-21 reduces the expression of RASA1, leading to the activation of the MAPK signaling pathway and the consequent proliferation of CRC cells [112]. The NF-κB pathway in *F. nucleatum*-infected CRC cells was also shown to downregulate the expression of miR-1322, leading to CCL20 activation, subsequently promoting macrophage infiltration, simultaneously inducing M2 macrophage polarization, and enhancing CRC metastasis [113]. Prometastatic behaviors of cells can also be mediated through the activation of the Wnt/β-catenin pathway. *F. nucleatum* infection stimulates CRC cells to generate exosomes enriched in miR-1246, miR-92b-3p, and miR-27a-3p that are internalized by non-infected cells, where they silence GSK3β, which regulates β-catenin turnover, thus increasing the cell migration ability and promoting tumor metastasis [114]. In association with *F. nucleatum*-induced CRC progression, miR-4474 and miR-4717 were found to be upregulated. Feng et al. suggest their ability to target and downregulate the CREB-binding protein (CBP), which plays a crucial role in Wnt/β-catenin signaling, one of the key pathways in CRC development [115] (Figure 3).

**Table 2:** miRNA with altered expression in specific bacterial-infection-positive CRC.

Bacteria	Altered miRNAs	miRNA Expression	Effect *	Reference
*F. nucleatum *	miR-4802; miR-18a*	down	▲ chemoresistance	[108]
	miR-21	up	▲ proliferation	[112]
	miR-34a; miR-135b	up	▲ inflammation; ▼ apoptosis	[27,109]
	miR-22-3p; miR-28-5p	down	▲ inflammation	[109]
	miR-4474; miR-4717	up	▲ CRC initiation	[115]
	miR-1322	down	▲ infiltration; ▲ polarization	[113]
	miR-1246; miR-92b-3p; miR-27a-3p	up	▲ migration	[114]
	miR-21; miR-17-5p; miR-155	up	NA	[111]
*E. coli*	miR-20a-5p	up	**▲** proliferation; **▲** tumor growth	[116]
	miR-30c; miR-130a	down	**▼** autophagy	[117]
*B. fragilis*	miR-155-5p; miR-200a-3p	down	**▲** tumor growth	[118]
	miR-149-3p	down	**▲** inflammation	[119]
*F. prausnitzii*	miR-92a	down	**▲** apoptosis; **▼** proliferation	[120]
	miR-203	up	**▲** apoptosis; **▼** proliferation; **▼** invasion	[121]

* Increased ▲/decreased ▼.

*E. coli* may also mediate CRC development by interacting with miRNAs. The majority of mucosa-associated *E. coli* isolated from CRC biopsies harbors the *pks* genomic island (*pks* + *E. coli*) responsible for the synthesis of genotoxic colibactin. Colibactin-producing *E. coli* increases cellular senescence characterized by the production of growth factors that promote the proliferation of uninfected cells and subsequently stimulate tumor growth. The underlying mechanisms involve *pks*+ *E. coli*-induced DNA damage, leading to an increased c-Myc expression. The proto-oncogene c-Myc encodes a transcription factor that binds to the miR-20a-5p promoter, thus upregulating its expression. miR-20a-5p targets SENP1 and silences a translation of this key protein that regulates p53 SUMOylation, an essential post-translational modification in eukaryotic cells [116]. Xing et al. discussed the mechanism of how adherent–invasive *E. coli*-infected epithelial cells may increase the proinflammatory response, consequently activating the NF-κB pathway and leading to miR-30c and miR-130a upregulation, which induces defective autophagy via ATG5 and ATG16L1 silencing, respectively [117].

Enterotoxigenic *B. fragilis* (ETBF) is a well-known tumor-inducing bacterium in the human gut that has been shown to induce tumor growth and promote inflammation by modulating lncRNA and miRNA molecules. ETBF-associated lncRNA1 (BFAL1) is highly expressed in CRC cells and provides an ability to competitively sponge miR-155-5p and miR-200a-3p molecules targeting the Ras homolog, the mTORC1 binding (*RHEB*) gene sequence. Thus, BFAL1 attenuates miRNA’s suppressive function and subsequently increases *RHEB* expression, leading to the activation of the downstream mTOR signaling pathway [118]. ETBF may also induce colorectal carcinogenesis by downregulating plasma exosomal miR-149-3p and further promoting the PHF5A-mediated RNA alternative splicing of KAT2A in CRC cells. Cao et al. suggest targeting the ETBF/miR-149-3p pathway as a promising approach to treat patients with intestinal inflammation and CRC with a high amount of ETBF [119].

*F. prausnitzii* is a well-known tumor-inducing bacterium, reported as one of the main butyrate producers in the human gut. Scientific evidence indicates that butyrate reduces the expression of c-Myc, thus inhibiting miR-92a transcription and leading to increased levels of p57 with a tumor-suppressive activity. These actions diminish CRC cell proliferation and stimulate apoptosis [120]. Sodium butyrate was also shown to induce CRC cell apoptosis and inhibit CRC cell proliferation, colony formation, and invasion through miR-203/NEDD9 cascade [121]. However, to our knowledge, the direct effect of *F. prausnitzii* on miR-203 expression in CRC cells has not been investigated yet.

### 4.2. Host-Derived miRNAs Affect Microbiome Gene Expression

The regulation of intestinal microbiota by host cells is, to a large extent, an unknown process. An analysis of recent studies by Rotschield et al. estimated the heritability of microbiota as between 1.8% and 8.1%, suggesting that the major influence on microbiome diversity and expression lies apart from diet on epigenetic modulation, possibly via miRNA [122]. The ability of host cells to reshape the intestinal microbiota via the miRNA pathway has been proven by Liu et al., who found that fecal miRNAs directly regulate specific bacterial gene expression and affect microbial growth in the gut [123]. The molecules miR-515-5p and miR-1226-5p were able to enter bacteria, co-localize with bacterial nucleic acids, and promote growth by altering the gene expression of CRC-associated bacterial species, such as *F. nucleatum* and *E. coli*, respectively. Animal studies have also demonstrated that particular host-produced miRNAs can suppress microbial growth. Knockout mice for CRC-associated miR-21 resulted in extensive growth of intestinal *Lactobacillus* sp., and a direct impact of human miR-21 on *Lactobacillus reuteri* growth reduction was also confirmed in vitro [124].

Since miRNAs (mainly derived from intestinal epithelial cells) are a normal constituent of human feces, they represent an important component of gut microbiota maintenance. However, different miRNAs provide different capacities to enter bacteria, leading to variation in their regulatory effects, so the mechanisms of the entry of miRNAs into bacteria and their subsequent processing require future investigation. Several studies have investigated the role of bacteria in CRC, but host–microbiome interaction ranges beyond bacterial species. However, to a lesser extent, the implication of the fungal microbiome in CRC development has also been discussed [125]. The pathogenic fungus *Candida albicans* was shown to communicate with human monocytes and induce the release of a human miRNA that promotes fungal growth. This mechanism represents an unexpected cross-species interaction and implies that the inhibition of specific miRNAs offers new possibilities for the treatment of human fungal infections [126].

### 4.3. Microbiome-Derived miRNAs Affect Host Gene Expression

It appears that microbiome-derived nucleic acids contribute to minute quantities of cell-free nucleic acids in human body fluids, indicating their potential as novel cancer biomarkers. They were detected in saliva, stool, blood, and plasma [127] and represent a new field for diagnostic research. One of the proposed mechanisms by which the gut microbiome influences the host transcriptome is the production of microbiota-derived snRNAs. Studies and tools that predict miRNA and miRNA-like molecules present in the genomes of microbiota are abundant, but only a fraction of them attempt to provide and verify the underlying molecular mechanisms.

In recent years, studies discussing viral miRNAs affecting host expression have emerged [128,129,130]. The production of viral miRNA has been mostly observed in herpesviruses such as Epstein Barr virus (EBV) [131,132,133,134], human cytomegalovirus [135,136], herpes simplex virus 1 and 2 [137,138,139,140], and Kaposi sarcoma-associated herpesvirus [133], as well as human immunodeficiency virus [141,142,143], Ebola virus [144], and Merkel cell polyomavirus [145]. The EBV genome encodes a cluster of 22 viral miRNAs called miR-BamHI-A rightward transcripts (miR-BARTs). Meng et al. report that EBV-miR-BART18-3p may promote CRC development by upregulating LDHA-mediated metabolic processes and the FASN-mediated de novo lipogenesis pathway. The ectopic expression of EBV-miR-BART18-3p leads to increased migration and invasion capacities of CRC cells in vitro and causes tumor metastasis in vivo. High EBV-miR-BART18-3p expression is closely associated with the pathological and advanced clinical stages of CRC, and thus it might serve as a potential diagnostic marker and therapeutic target for CRC [146].

Considering non-bacteria microbiomes, fungi producing miRNA to interact with hosts are poorly explored. To our knowledge, there is no evidence for a direct interaction between gut fungi miRNAs and human gene expression machinery, but several records confirm the existence of cross-kingdom small-RNA-interference pathways that can affect host gene expression. The fungal miRNA-like molecule bba-milR1 produced by *Beauveria bassiana* has been found to suppress *Anopheles stephensi* immunity to enable fungal penetration of the mosquito integument [147]. The plant pathogenic fungus *Fusarium oxysporum* has been proven to regulate gene expression in chickpea via miRNA [148]. Fungal miRNAs were also detected in a mutualistic interaction between the mycorrhizal fungus *Pisolithus microcarpus* producing Pmic_miR-8, which allows the fungus to integrate deeper into *Eucalyptus grandis*’ tissues and positively affects the maintenance of its mycorrhizal roots [149].

Studies generally discuss the mode of host miRNA expression regulated via the MyD88-dependent pathway or gut-microbiome-derived metabolites. However, there is scarce evidence for an interaction between gut microbial snRNAs and human gene expression machinery. Although miRNAs were found in various species of plants, animals, and viruses, little is known about miRNA-sized RNAs (msRNAs) in prokaryotes. The surrounding msRNA sequences in bacteria have been shown to have a structure similar to precursor miRNA molecules, and supporting analyses suggest that msRNA may be processed from longer RNAs in a way similar to miRNA maturation [150]. On the other hand, miRNAs act through RNA-induced silencing complex (RISC), which does not exist in bacteria. However, bacterial Hfq RNA chaperone mediates and stabilizes RNA–RNA interactions and provides a similar role as RISC [151]. Only a few studies have observed how bacterial msRNAs or miRNA-like molecules interact with macro-organisms. *Salmonella enteritidis* produces miRNA-like RNAs Sal-1 by exploiting host AGO2 protein in infected cells. The production of Sal-1 promotes the intracellular survival of *S. enteritidis* and helps to evade the host immune system [152]. Furuse et al. observed that miRNA-like RNA MM-H is produced by *Mycobacterium marinum* in infected macrophage cells, but they were unable to investigate its function in *M. marinum*-infected cells [153]. Small secretable RNAs were detected in the periodontal pathogens *Aggregatibacter actinomycetemcomitans*, *Porphyromonas gingivalis*, and *Treponema denticola*. Those small RNAs were exported in extracellular vesicles and suppressed the production of IL-5, IL-13, and IL-15 in T-cells [154]. These studies underline that further research is needed to understand how microbiomes are capable of interacting and affecting host expression by the means of miRNA-like molecules.

## 5. miRNAs and Gut Microbiota Therapeutic Implications

Due to the evident relationship between miRNAs and the development of colorectal cancer, further research focused on the possibilities of probiotics and targeted nutrition as novel therapeutic concepts must be conducted. It is well known that probiotics can regulate the expression of intestinal miRNAs [155] and that the host’s diet affects the variety and abundance of the microbiota [156]. Among the probiotics studied to alter miRNA expression in colorectal cancer, *Lactobacillus acidophilus* and *Bifidobacterium bifidum* showed potential to upregulate miRNAs with tumor-suppressive properties and downregulate oncogene-associated miRNAs [157]. Moreover, animal studies have demonstrated the ability of *Bifidobacterium longum* to inhibit colorectal cancer by inducing miR-145 and miR-15a tumor-suppressive properties [158]. miRNAs identified as either downregulated or upregulated in CRC patients (Table S1) can provide potential therapeutic targets for miRNA-based therapy using miRNA mimics or antisense RNAs to restore or reduce endogenous miRNA levels, respectively. Among the main advantages of miRNA-based therapies might be their lower toxicity due to their natural occurrence in the intestine compared to currently used chemical substances [159]. However, the lack of miRNA specificity towards the target [160] and problems associated with the delivery of therapeutic agents to cells of interest [161] currently limit their therapeutic use in clinical practice.

## 6. Conclusions

Over the past decade, gut microbiota dysbiosis has been linked to many health disorders, including CRC. Since miRNAs were shown to regulate microbiome composition and play a critical role in intestinal homeostasis, specific microbiome–miRNA correlations can serve as non-invasive diagnostic biomarkers for CRC. Uncovering the interactions between CRC-associated miRNAs and microbiome entities such as bacteria, phages, viruses, or fungi expands our understanding of CRC cell biology and could help to identify novel therapeutic targets. Such cross-kingdom communication supports the idea of targeting miRNAs to aid in manipulating the balance of the microbiota. However, the mechanism of miRNA-mediated colorectal tumorigenesis considering microbial composition is still poorly understood, and for some microbial representatives it is not at all understood. Thus, further studies are necessary to elucidate the benefit of this potential non-invasive multi-omics biomarker approach.

## Data Availability

Not applicable.

## Supplementary Materials

The following supporting information can be downloaded at: https://www.mdpi.com/article/10.3390/ijms241310520/s1. References [19,20,26,27,43,48,49,162,163,164,165,166,167,168,169,170,171,172,173,174,175,176,177,178,179,180,181,182,183,184,185,186,187,188,189,190,191,192,193,194,195,196,197,198,199,200,201,202,203,204,205,206,207,208,209,210,211,212,213,214,215,216,217,218,219,220,221,222,223,224,225,226,227,228,229,230,231,232,233,234,235,236,237,238,239,240,241,242,243,244,245,246] are cited in the Supplementary Materials

## References

[1] Tepus M., Yau T.O. (2020). Non-Invasive Colorectal Cancer Screening: An Overview. Gastrointest. Tumors.

[2] Issa I.A., Noureddine M. (2017). Colorectal Cancer Screening: An Updated Review of the Available Options. World J. Gastroenterol..

[3] Halloran S.P., Launoy G., Zappa M. (2012). International Agency for Research on Cancer European Guidelines for Quality Assurance in Colorectal Cancer Screening and Diagnosis. First Edition—Faecal Occult Blood Testing. Endoscopy.

[4] Davidson K.W., Barry M.J., Mangione C.M., Cabana M., Caughey A.B., Davis E.M., Donahue K.E., Doubeni C.A., Krist A.H., US Preventive Services Task Force (2021). Screening for Colorectal Cancer: US Preventive Services Task Force Recommendation Statement. JAMA.

[5] de Klaver W., Wisse P.H.A., van Wifferen F., Bosch L.J.W., Jimenez C.R., van der Hulst R.W.M., Fijneman R.J.A., Kuipers E.J., Greuter M.J.E., Carvalho B. (2021). Clinical Validation of a Multitarget Fecal Immunochemical Test for Colorectal Cancer Screening: A Diagnostic Test Accuracy Study. Ann. Intern. Med..

[6] Gies A., Cuk K., Schrotz-King P., Brenner H. (2018). Direct Comparison of Diagnostic Performance of 9 Quantitative Fecal Immunochemical Tests for Colorectal Cancer Screening. Gastroenterology.

[7] van Dam L., Kuipers E.J., van Leerdam M.E. (2010). Performance Improvements of Stool-Based Screening Tests. Best Pract. Res. Clin. Gastroenterol..

[8] Kaminski M.F., Robertson D.J., Senore C., Rex D.K. (2020). Optimizing the Quality of Colorectal Cancer Screening Worldwide. Gastroenterology.

[9] Schliemann D., Matovu N., Ramanathan K., Muñoz-Aguirre P., O’Neill C., Kee F., Su T.T., Donnelly M. (2020). Implementation of Colorectal Cancer Screening Interventions in Low-Income and Middle-Income Countries: A Scoping Review Protocol. BMJ Open.

[10] Schreuders E.H., Ruco A., Rabeneck L., Schoen R.E., Sung J.J.Y., Young G.P., Kuipers E.J. (2015). Colorectal Cancer Screening: A Global Overview of Existing Programmes. Gut.

[11] Toden S., Goel A. (2022). Non-Coding RNAs as Liquid Biopsy Biomarkers in Cancer. Br. J. Cancer.

[12] Zygulska A.L., Pierzchalski P. (2022). Novel Diagnostic Biomarkers in Colorectal Cancer. Int. J. Mol. Sci..

[13] Ali A., Ara A., Kashyap M.K. (2022). Gut Microbiota: Role and Association with Tumorigenesis in Different Malignancies. Mol. Biol. Rep..

[14] O’Brien J., Hayder H., Zayed Y., Peng C. (2018). Overview of MicroRNA Biogenesis, Mechanisms of Actions, and Circulation. Front. Endocrinol..

[15] Romano G., Veneziano D., Acunzo M., Croce C.M. (2017). Small Non-Coding RNA and Cancer. Carcinogenesis.

[16] Pös O., Biró O., Szemes T., Nagy B. (2018). Circulating Cell-Free Nucleic Acids: Characteristics and Applications. Eur. J. Hum. Genet..

[17] Rounge T.B., Lauritzen M., Langseth H., Enerly E., Lyle R., Gislefoss R.E. (2015). microRNA Biomarker Discovery and High-Throughput DNA Sequencing Are Possible Using Long-Term Archived Serum Samples. Cancer Epidemiol. Biomark. Prev..

[18] Chen X., Ba Y., Ma L., Cai X., Yin Y., Wang K., Guo J., Zhang Y., Chen J., Guo X. (2008). Characterization of microRNAs in Serum: A Novel Class of Biomarkers for Diagnosis of Cancer and Other Diseases. Cell Res..

[19] Ng E.K.O., Chong W.W.S., Jin H., Lam E.K.Y., Shin V.Y., Yu J., Poon T.C.W., Ng S.S.M., Sung J.J.Y. (2009). Differential Expression of microRNAs in Plasma of Patients with Colorectal Cancer: A Potential Marker for Colorectal Cancer Screening. Gut.

[20] Ding L., Lan Z., Xiong X., Ao H., Feng Y., Gu H., Yu M., Cui Q. (2018). The Dual Role of MicroRNAs in Colorectal Cancer Progression. Int. J. Mol. Sci..

[21] Esquela-Kerscher A., Slack F.J. (2006). Oncomirs-microRNAs with a Role in Cancer. Nat. Rev. Cancer.

[22] Bautista-Sánchez D., Arriaga-Canon C., Pedroza-Torres A., De La Rosa-Velázquez I.A., González-Barrios R., Contreras-Espinosa L., Montiel-Manríquez R., Castro-Hernández C., Fragoso-Ontiveros V., Álvarez-Gómez R.M. (2020). The Promising Role of miR-21 as a Cancer Biomarker and Its Importance in RNA-Based Therapeutics. Mol. Ther. Nucleic Acids.

[23] (2018). MicroRNAs as Potential Liquid Biopsy Biomarkers in Colorectal Cancer: A Systematic Review. Biochim. Biophys. Acta (BBA)-Rev. Cancer.

[24] Kanaan Z., Rai S.N., Eichenberger M.R., Roberts H., Keskey B., Pan J., Galandiuk S. (2012). Plasma miR-21: A Potential Diagnostic Marker of Colorectal Cancer. Ann. Surg..

[25] Toiyama Y., Takahashi M., Hur K., Nagasaka T., Tanaka K., Inoue Y., Kusunoki M., Boland C.R., Goel A. (2013). Serum miR-21 as a Diagnostic and Prognostic Biomarker in Colorectal Cancer. J. Natl. Cancer Inst..

[26] Wu C.W., Ng S.C., Dong Y., Tian L., Ng S.S.M., Leung W.W., Law W.T., Yau T.O., Chan F.K.L., Sung J.J.Y. (2014). Identification of microRNA-135b in Stool as a Potential Noninvasive Biomarker for Colorectal Cancer and Adenoma. Clin. Cancer Res..

[27] Wang H., Wang X., Zhang H., Deng T., Liu R., Liu Y., Li H., Bai M., Ning T., Wang J. (2021). The HSF1/miR-135b-5p Axis Induces Protective Autophagy to Promote Oxaliplatin Resistance through the MUL1/ULK1 Pathway in Colorectal Cancer. Oncogene.

[28] Urbich C., Kuehbacher A., Dimmeler S. (2008). Role of microRNAs in Vascular Diseases, Inflammation, and Angiogenesis. Cardiovasc. Res..

[29] Olaru A.V., Yamanaka S., Vazquez C., Mori Y., Cheng Y., Abraham J.M., Bayless T.M., Harpaz N., Selaru F.M., Meltzer S.J. (2013). MicroRNA-224 Negatively Regulates p21 Expression during Late Neoplastic Progression in Inflammatory Bowel Disease. Inflamm. Bowel Dis..

[30] Zhang X., Ai F., Li X., Tian L., Wang X., Shen S., Liu F. (2017). MicroRNA-34a Suppresses Colorectal Cancer Metastasis by Regulating Notch Signaling. Oncol. Lett..

[31] Hosseini M., Baghaei K., Amani D., Ebtekar M. (2021). Tumor-Derived Exosomes Encapsulating miR-34a Promote Apoptosis and Inhibit Migration and Tumor Progression of Colorectal Cancer Cells under in Vitro Condition. Daru.

[32] Gao X., Xu W., Lu T., Zhou J., Ge X., Hua D. (2018). MicroRNA-142-3p Promotes Cellular Invasion of Colorectal Cancer Cells by Activation of RAC1. Technol. Cancer Res. Treat..

[33] Xie N., Meng Q., Zhang Y., Luo Z., Xue F., Liu S., Li Y., Huang Y. (2021). MicroRNA-142-3p Suppresses Cell Proliferation, Invasion and Epithelial-to-mesenchymal Transition via RAC1-ERK1/2 Signaling in Colorectal Cancer. Mol. Med. Rep..

[34] Liu J., Chen Z., Xiang J., Gu X. (2018). MicroRNA-155 Acts as a Tumor Suppressor in Colorectal Cancer by Targeting CTHRC1. Oncol. Lett..

[35] Zhang G.-J., Xiao H.-X., Tian H.-P., Liu Z.-L., Xia S.-S., Zhou T. (2013). Upregulation of microRNA-155 Promotes the Migration and Invasion of Colorectal Cancer Cells through the Regulation of Claudin-1 Expression. Int. J. Mol. Med..

[36] Wan Y.-C., Li T., Han Y.-D., Zhang H.-Y., Lin H., Zhang B. (2022). [Retracted] MicroRNA-155 Enhances the Activation of Wnt/β-catenin Signaling in Colorectal Carcinoma by Suppressing HMG-box Transcription Factor 1. Mol. Med. Rep..

[37] Jorgensen B.G., Ro S. (2022). MicroRNAs and “Sponging” Competitive Endogenous RNAs Dysregulated in Colorectal Cancer: Potential as Noninvasive Biomarkers and Therapeutic Targets. Int. J. Mol. Sci..

[38] Liu J., Liu F., Li X., Song X., Zhou L., Jie J. (2017). Screening Key Genes and miRNAs in Early-Stage Colon Adenocarcinoma by RNA-Sequencing. Tumour Biol..

[39] Zekri A.-R.N., Youssef A.S.E.-D., Lotfy M.M., Gabr R., Ahmed O.S., Nassar A., Hussein N., Omran D., Medhat E., Eid S. (2016). Circulating Serum miRNAs as Diagnostic Markers for Colorectal Cancer. PLoS ONE.

[40] Wang Y.-N., Chen Z.-H., Chen W.-C. (2017). Novel Circulating microRNAs Expression Profile in Colon Cancer: A Pilot Study. Eur. J. Med. Res..

[41] Maminezhad H., Ghanadian S., Pakravan K., Razmara E., Rouhollah F., Mossahebi-Mohammadi M., Babashah S. (2020). A Panel of Six-Circulating miRNA Signature in Serum and Its Potential Diagnostic Value in Colorectal Cancer. Life Sci..

[42] Herreros-Villanueva M., Duran-Sanchon S., Martín A.C., Pérez-Palacios R., Vila-Navarro E., Marcuello M., Diaz-Centeno M., Cubiella J., Diez M.S., Bujanda L. (2019). Plasma MicroRNA Signature Validation for Early Detection of Colorectal Cancer. Clin. Transl. Gastroenterol..

[43] Eslamizadeh S., Heidari M., Agah S., Faghihloo E., Ghazi H., Mirzaei A., Akbari A. (2018). The Role of MicroRNA Signature as Diagnostic Biomarkers in Different Clinical Stages of Colorectal Cancer. Cell J..

[44] Toiyama Y., Hur K., Tanaka K., Inoue Y., Kusunoki M., Boland C.R., Goel A. (2014). Serum miR-200c Is a Novel Prognostic and Metastasis-Predictive Biomarker in Patients with Colorectal Cancer. Ann. Surg..

[45] Nakatsu G., Li X., Zhou H., Sheng J., Wong S.H., Wu W.K.K., Ng S.C., Tsoi H., Dong Y., Zhang N. (2015). Gut Mucosal Microbiome across Stages of Colorectal Carcinogenesis. Nat. Commun..

[46] Yachida S., Mizutani S., Shiroma H., Shiba S., Nakajima T., Sakamoto T., Watanabe H., Masuda K., Nishimoto Y., Kubo M. (2019). Metagenomic and Metabolomic Analyses Reveal Distinct Stage-Specific Phenotypes of the Gut Microbiota in Colorectal Cancer. Nat. Med..

[47] Phua L.C., Chue X.P., Koh P.K., Cheah P.Y., Chan E.C.Y., Ho H.K. (2014). Global Fecal microRNA Profiling in the Identification of Biomarkers for Colorectal Cancer Screening among Asians. Oncol. Rep..

[48] Chen B., Xia Z., Deng Y.-N., Yang Y., Zhang P., Zhu H., Xu N., Liang S. (2019). Emerging microRNA Biomarkers for Colorectal Cancer Diagnosis and Prognosis. Open Biol..

[49] Koga Y., Yamazaki N., Yamamoto Y., Yamamoto S., Saito N., Kakugawa Y., Otake Y., Matsumoto M., Matsumura Y. (2013). Fecal miR-106a Is a Useful Marker for Colorectal Cancer Patients with False-Negative Results in Immunochemical Fecal Occult Blood Test. Cancer Epidemiol. Biomark. Prev..

[50] Szilágyi M., Pös O., Márton É., Buglyó G., Soltész B., Keserű J., Penyige A., Szemes T., Nagy B. (2020). Circulating Cell-Free Nucleic Acids: Main Characteristics and Clinical Application. Int. J. Mol. Sci..

[51] Faraldi M., Gomarasca M., Banfi G., Lombardi G. (2018). Free Circulating miRNAs Measurement in Clinical Settings: The Still Unsolved Issue of the Normalization. Adv. Clin. Chem..

[52] Faraldi M., Gomarasca M., Sansoni V., Perego S., Banfi G., Lombardi G. (2019). Normalization Strategies Differently Affect Circulating miRNA Profile Associated with the Training Status. Sci. Rep..

[53] McDonald J.S., Milosevic D., Reddi H.V., Grebe S.K., Algeciras-Schimnich A. (2011). Analysis of Circulating microRNA: Preanalytical and Analytical Challenges. Clin. Chem..

[54] Becker C., Hammerle-Fickinger A., Riedmaier I., Pfaffl M.W. (2010). mRNA and microRNA Quality Control for RT-qPCR Analysis. Methods.

[55] Mestdagh P., Van Vlierberghe P., De Weer A., Muth D., Westermann F., Speleman F., Vandesompele J. (2009). A Novel and Universal Method for microRNA RT-qPCR Data Normalization. Genome Biol..

[56] Pös Z., Pös O., Styk J., Mocova A., Strieskova L., Budis J., Kadasi L., Radvanszky J., Szemes T. (2020). Technical and Methodological Aspects of Cell-Free Nucleic Acids Analyzes. Int. J. Mol. Sci..

[57] Scarpellini E., Ianiro G., Attili F., Bassanelli C., De Santis A., Gasbarrini A. (2015). The Human Gut Microbiota and Virome: Potential Therapeutic Implications. Dig. Liver Dis..

[58] Stearns J.C., Lynch M.D.J., Senadheera D.B., Tenenbaum H.C., Goldberg M.B., Cvitkovitch D.G., Croitoru K., Moreno-Hagelsieb G., Neufeld J.D. (2011). Bacterial Biogeography of the Human Digestive Tract. Sci. Rep..

[59] Rajilić-Stojanović M., Smidt H., de Vos W.M. (2007). Diversity of the Human Gastrointestinal Tract Microbiota Revisited. Environ. Microbiol..

[60] Ursell L.K., Metcalf J.L., Parfrey L.W., Knight R. (2012). Defining the Human Microbiome. Nutr. Rev..

[61] Geuking M.B., Köller Y., Rupp S., McCoy K.D. (2014). The Interplay between the Gut Microbiota and the Immune System. Gut Microbes.

[62] Yadav D., Ghosh T.S., Mande S.S. (2016). Global Investigation of Composition and Interaction Networks in Gut Microbiomes of Individuals Belonging to Diverse Geographies and Age-Groups. Gut Pathog..

[63] Yatsunenko T., Rey F.E., Manary M.J., Trehan I., Dominguez-Bello M.G., Contreras M., Magris M., Hidalgo G., Baldassano R.N., Anokhin A.P. (2012). Human Gut Microbiome Viewed across Age and Geography. Nature.

[64] Engen P.A., Green S.J., Voigt R.M., Forsyth C.B., Keshavarzian A. (2015). The Gastrointestinal Microbiome: Alcohol Effects on the Composition of Intestinal Microbiota. Alcohol Res..

[65] Xu Z., Knight R. (2015). Dietary Effects on Human Gut Microbiome Diversity. Br. J. Nutr..

[66] Gupta V.K., Paul S., Dutta C. (2017). Geography, Ethnicity or Subsistence-Specific Variations in Human Microbiome Composition and Diversity. Front. Microbiol..

[67] Sheflin A.M., Whitney A.K., Weir T.L. (2014). Cancer-Promoting Effects of Microbial Dysbiosis. Curr. Oncol. Rep..

[68] Mármol I., Sánchez-de-Diego C., Pradilla Dieste A., Cerrada E., Rodriguez Yoldi M.J. (2017). Colorectal Carcinoma: A General Overview and Future Perspectives in Colorectal Cancer. Int. J. Mol. Sci..

[69] Turnbaugh P.J., Ley R.E., Mahowald M.A., Magrini V., Mardis E.R., Gordon J.I. (2006). An Obesity-Associated Gut Microbiome with Increased Capacity for Energy Harvest. Nature.

[70] Chung H., Pamp S.J., Hill J.A., Surana N.K., Edelman S.M., Troy E.B., Reading N.C., Villablanca E.J., Wang S., Mora J.R. (2012). Gut Immune Maturation Depends on Colonization with a Host-Specific Microbiota. Cell.

[71] Rebersek M. (2021). Gut Microbiome and Its Role in Colorectal Cancer. BMC Cancer.

[72] Tjalsma H., Boleij A., Marchesi J.R., Dutilh B.E. (2012). A Bacterial Driver-Passenger Model for Colorectal Cancer: Beyond the Usual Suspects. Nat. Rev. Microbiol..

[73] Ley R.E., Hamady M., Lozupone C., Turnbaugh P.J., Ramey R.R., Bircher J.S., Schlegel M.L., Tucker T.A., Schrenzel M.D., Knight R. (2008). Evolution of Mammals and Their Gut Microbes. Science.

[74] Davenport E.R., Mizrahi-Man O., Michelini K., Barreiro L.B., Ober C., Gilad Y. (2014). Seasonal Variation in Human Gut Microbiome Composition. PLoS ONE.

[75] Sankar S.A., Lagier J.-C., Pontarotti P., Raoult D., Fournier P.-E. (2015). The Human Gut Microbiome, a Taxonomic Conundrum. Syst. Appl. Microbiol..

[76] Luan C., Xie L., Yang X., Miao H., Lv N., Zhang R., Xiao X., Hu Y., Liu Y., Wu N. (2015). Dysbiosis of Fungal Microbiota in the Intestinal Mucosa of Patients with Colorectal Adenomas. Sci. Rep..

[77] Castellarin M., Warren R.L., Freeman J.D., Dreolini L., Krzywinski M., Strauss J., Barnes R., Watson P., Allen-Vercoe E., Moore R.A. (2012). Fusobacterium Nucleatum Infection Is Prevalent in Human Colorectal Carcinoma. Genome Res..

[78] Feng Q., Liang S., Jia H., Stadlmayr A., Tang L., Lan Z., Zhang D., Xia H., Xu X., Jie Z. (2015). Gut Microbiome Development along the Colorectal Adenoma-Carcinoma Sequence. Nat. Commun..

[79] Yu J., Feng Q., Wong S.H., Zhang D., Liang Q.Y., Qin Y., Tang L., Zhao H., Stenvang J., Li Y. (2017). Metagenomic Analysis of Faecal Microbiome as a Tool towards Targeted Non-Invasive Biomarkers for Colorectal Cancer. Gut.

[80] Flemer B., Lynch D.B., Brown J.M.R., Jeffery I.B., Ryan F.J., Claesson M.J., O’Riordain M., Shanahan F., O’Toole P.W. (2017). Tumour-Associated and Non-Tumour-Associated Microbiota in Colorectal Cancer. Gut.

[81] Seki H., Shiohara M., Matsumura T., Miyagawa N., Tanaka M., Komiyama A., Kurata S. (2003). Prevention of Antibiotic-Associated Diarrhea in Children by Clostridium Butyricum MIYAIRI. Pediatr. Int..

[82] Corrêa N.B.O., Péret Filho L.A., Penna F.J., Lima F.M.L.S., Nicoli J.R. (2005). A Randomized Formula Controlled Trial of Bifidobacterium Lactis and Streptococcus Thermophilus for Prevention of Antibiotic-Associated Diarrhea in Infants. J. Clin. Gastroenterol..

[83] Cheng Y., Ling Z., Li L. (2020). The Intestinal Microbiota and Colorectal Cancer. Front. Immunol..

[84] Wong S.H., Yu J. (2019). Gut Microbiota in Colorectal Cancer: Mechanisms of Action and Clinical Applications. Nat. Rev. Gastroenterol. Hepatol..

[85] Worthley D.L., Cole S.R., Esterman A., Mehaffey S., Roosa N.M., Smith A., Turnbull D., Young G.P. (2006). Screening for Colorectal Cancer by Faecal Occult Blood Test: Why People Choose to Refuse. Intern. Med. J..

[86] Narunsky-Haziza L., Sepich-Poore G.D., Livyatan I., Asraf O., Martino C., Nejman D., Gavert N., Stajich J.E., Amit G., González A. (2022). Pan-Cancer Analyses Reveal Cancer-Type-Specific Fungal Ecologies and Bacteriome Interactions. Cell.

[87] Dohlman A.B., Klug J., Mesko M., Gao I.H., Lipkin S.M., Shen X., Iliev I.D. (2022). A Pan-Cancer Mycobiome Analysis Reveals Fungal Involvement in Gastrointestinal and Lung Tumors. Cell.

[88] Haghi F., Goli E., Mirzaei B., Zeighami H. (2019). The Association between Fecal Enterotoxigenic B. Fragilis with Colorectal Cancer. BMC Cancer.

[89] Cheng W.T., Kantilal H.K., Davamani F. (2020). The Mechanism of Toxin Contributes to Colon Cancer Formation. Malays. J. Med. Sci..

[90] Wu S., Morin P.J., Maouyo D., Sears C.L. (2003). Bacteroides Fragilis Enterotoxin Induces c-Myc Expression and Cellular Proliferation. Gastroenterology.

[91] Zhou Y., He H., Xu H., Li Y., Li Z., Du Y., He J., Zhou Y., Wang H., Nie Y. (2016). Association of Oncogenic Bacteria with Colorectal Cancer in South China. Oncotarget.

[92] Balish E., Warner T. (2002). Enterococcus Faecalis Induces Inflammatory Bowel Disease in Interleukin-10 Knockout Mice. Am. J. Pathol..

[93] Kim S.C., Tonkonogy S.L., Albright C.A., Tsang J., Balish E.J., Braun J., Huycke M.M., Sartor R.B. (2005). Variable Phenotypes of Enterocolitis in Interleukin 10-Deficient Mice Monoassociated with Two Different Commensal Bacteria. Gastroenterology.

[94] Iyadorai T., Mariappan V., Vellasamy K.M., Wanyiri J.W., Roslani A.C., Lee G.K., Sears C., Vadivelu J. (2020). Prevalence and Association of Pks+ Escherichia Coli with Colorectal Cancer in Patients at the University Malaya Medical Centre, Malaysia. PLoS ONE.

[95] Taieb F., Petit C., Nougayrède J.-P., Oswald E. (2016). The Enterobacterial Genotoxins: Cytolethal Distending Toxin and Colibactin. EcoSal Plus.

[96] Zhao H., Ming T., Tang S., Ren S., Yang H., Liu M., Tao Q., Xu H. (2022). Wnt Signaling in Colorectal Cancer: Pathogenic Role and Therapeutic Target. Mol. Cancer.

[97] Rubinstein M.R., Wang X., Liu W., Hao Y., Cai G., Han Y.W. (2013). Fusobacterium Nucleatum Promotes Colorectal Carcinogenesis by Modulating E-Cadherin/β-Catenin Signaling via Its FadA Adhesin. Cell Host Microbe.

[98] Kostic A.D., Chun E., Robertson L., Glickman J.N., Gallini C.A., Michaud M., Clancy T.E., Chung D.C., Lochhead P., Hold G.L. (2013). Fusobacterium Nucleatum Potentiates Intestinal Tumorigenesis and Modulates the Tumor-Immune Microenvironment. Cell Host Microbe.

[99] Uchino Y., Goto Y., Konishi Y., Tanabe K., Toda H., Wada M., Kita Y., Beppu M., Mori S., Hijioka H. (2021). Colorectal Cancer Patients Have Four Specific Bacterial Species in Oral and Gut Microbiota in Common-A Metagenomic Comparison with Healthy Subjects. Cancers.

[100] Abdulamir A.S., Hafidh R.R., Abu Bakar F. (2011). The Association of Streptococcus Bovis/gallolyticus with Colorectal Tumors: The Nature and the Underlying Mechanisms of Its Etiological Role. J. Exp. Clin. Cancer Res..

[101] Aymeric L., Donnadieu F., Mulet C., du Merle L., Nigro G., Saffarian A., Bérard M., Poyart C., Robine S., Regnault B. (2018). Colorectal Cancer Specific Conditions Promote Gut Colonization. Proc. Natl. Acad. Sci. USA.

[102] Zhu Z., Huang J., Li X., Xing J., Chen Q., Liu R., Hua F., Qiu Z., Song Y., Bai C. (2020). Gut Microbiota Regulate Tumor Metastasis via circRNA/miRNA Networks. Gut Microbes.

[103] Fan S., Xing J., Jiang Z., Zhang Z., Zhang H., Wang D., Tang D. (2022). Effects of Long Non-Coding RNAs Induced by the Gut Microbiome on Regulating the Development of Colorectal Cancer. Cancers.

[104] Nikolaieva N., Sevcikova A., Omelka R., Martiniakova M., Mego M., Ciernikova S. (2022). Gut Microbiota-MicroRNA Interactions in Intestinal Homeostasis and Cancer Development. Microorganisms.

[105] Anfossi S., Calin G.A. (2020). Gut Microbiota: A New Player in Regulating Immune- and Chemo-Therapy Efficacy. Cancer Drug Resist.

[106] Tarallo S., Ferrero G., Gallo G., Francavilla A., Clerico G., Realis Luc A., Manghi P., Thomas A.M., Vineis P., Segata N. (2019). Altered Fecal Small RNA Profiles in Colorectal Cancer Reflect Gut Microbiome Composition in Stool Samples. Msystems.

[107] Loktionov A. (2020). Biomarkers for Detecting Colorectal Cancer Non-Invasively: DNA, RNA or Proteins?. World J. Gastrointest. Oncol..

[108] Yu T., Guo F., Yu Y., Sun T., Ma D., Han J., Qian Y., Kryczek I., Sun D., Nagarsheth N. (2017). Fusobacterium Nucleatum Promotes Chemoresistance to Colorectal Cancer by Modulating Autophagy. Cell.

[109] Proença M.A., Biselli J.M., Succi M., Severino F.E., Berardinelli G.N., Caetano A., Reis R.M., Hughes D.J., Silva A.E. (2018). Relationship between, Inflammatory Mediators and microRNAs in Colorectal Carcinogenesis. World J. Gastroenterol..

[110] Li P., Fan J.-B., Gao Y., Zhang M., Zhang L., Yang N., Zhao X. (2016). miR-135b-5p Inhibits LPS-Induced TNFα Production via Silencing AMPK Phosphatase Ppm1e. Oncotarget.

[111] Bostanshirin N., Hajikhani B., Vaezi A.A., Kermanian F., Sameni F., Yaslianifard S., Goudarzi M., Dadashi M. (2023). Association between Colorectal Cancer and Expression Levels of miR-21, miR-17-5P, miR-155 Genes and the Presence of Fusobacterium Nucleatum in Biopsy Samples Obtained from Iranian Patients. Infect. Agent. Cancer.

[112] Yang Y., Weng W., Peng J., Hong L., Yang L., Toiyama Y., Gao R., Liu M., Yin M., Pan C. (2017). Fusobacterium Nucleatum Increases Proliferation of Colorectal Cancer Cells and Tumor Development in Mice by Activating Toll-Like Receptor 4 Signaling to Nuclear Factor-κB, and Up-Regulating Expression of MicroRNA-21. Gastroenterology.

[113] Xu C., Fan L., Lin Y., Shen W., Qi Y., Zhang Y., Chen Z., Wang L., Long Y., Hou T. (2021). Promotes Colorectal Cancer Metastasis through miR-1322/CCL20 Axis and M2 Polarization. Gut Microbes.

[114] Guo S., Chen J., Chen F., Zeng Q., Liu W.-L., Zhang G. (2020). Exosomes Derived from -Infected Colorectal Cancer Cells Facilitate Tumour Metastasis by Selectively Carrying miR-1246/92b-3p/27a-3p and CXCL16. Gut.

[115] Feng Y.-Y., Zeng D.-Z., Tong Y.-N., Lu X.-X., Dun G.-D., Tang B., Zhang Z.-J., Ye X.-L., Li Q., Xie J.-P. (2019). Alteration of microRNA-4474/4717 Expression and CREB-Binding Protein in Human Colorectal Cancer Tissues Infected with Fusobacterium Nucleatum. PLoS ONE.

[116] Dalmasso G., Cougnoux A., Delmas J., Darfeuille-Michaud A., Bonnet R. (2014). The Bacterial Genotoxin Colibactin Promotes Colon Tumor Growth by Modifying the Tumor Microenvironment. Gut Microbes.

[117] Xing J., Liao Y., Zhang H., Zhang W., Zhang Z., Zhang J., Wang D., Tang D. (2022). Impacts of MicroRNAs Induced by the Gut Microbiome on Regulating the Development of Colorectal Cancer. Front. Cell. Infect. Microbiol..

[118] Bao Y., Tang J., Qian Y., Sun T., Chen H., Chen Z., Sun D., Zhong M., Chen H., Hong J. (2019). Long Noncoding RNA BFAL1 Mediates Enterotoxigenic Bacteroides Fragilis-Related Carcinogenesis in Colorectal Cancer via the RHEB/mTOR Pathway. Cell Death Dis..

[119] Cao Y., Wang Z., Yan Y., Ji L., He J., Xuan B., Shen C., Ma Y., Jiang S., Ma D. (2021). Enterotoxigenic Bacteroidesfragilis Promotes Intestinal Inflammation and Malignancy by Inhibiting Exosome-Packaged miR-149-3p. Gastroenterology.

[120] Hu S., Liu L., Chang E.B., Wang J.-Y., Raufman J.-P. (2015). Butyrate Inhibits pro-Proliferative miR-92a by Diminishing c-Myc-Induced miR-17-92a Cluster Transcription in Human Colon Cancer Cells. Mol. Cancer.

[121] Han R., Sun Q., Wu J., Zheng P., Zhao G. (2016). Sodium Butyrate Upregulates miR-203 Expression to Exert Anti-Proliferation Effect on Colorectal Cancer Cells. Cell. Physiol. Biochem..

[122] Rothschild D., Weissbrod O., Barkan E., Kurilshikov A., Korem T., Zeevi D., Costea P.I., Godneva A., Kalka I.N., Bar N. (2018). Environment Dominates over Host Genetics in Shaping Human Gut Microbiota. Nature.

[123] Liu S., da Cunha A.P., Rezende R.M., Cialic R., Wei Z., Bry L., Comstock L.E., Gandhi R., Weiner H.L. (2016). The Host Shapes the Gut Microbiota via Fecal MicroRNA. Cell Host Microbe.

[124] Santos A.A., Afonso M.B., Ramiro R.S., Pires D., Pimentel M., Castro R.E., Rodrigues C.M.P. (2020). Host miRNA-21 Promotes Liver Dysfunction by Targeting Small Intestinal in Mice. Gut Microbes.

[125] Anandakumar A., Pellino G., Tekkis P., Kontovounisios C. (2019). Fungal Microbiome in Colorectal Cancer: A Systematic Review. Updates Surg..

[126] Halder L.D., Babych S., Palme D.I., Mansouri-Ghahnavieh E., Ivanov L., Ashonibare V., Langenhorst D., Prusty B., Rambach G., Wich M. (2021). Candida Albicans Induces Cross-Kingdom miRNA Trafficking in Human Monocytes To Promote Fungal Growth. MBio.

[127] Zozaya-Valdés E., Wong S.Q., Raleigh J., Hatzimihalis A., Ftouni S., Papenfuss A.T., Sandhu S., Dawson M.A., Dawson S.-J. (2021). Detection of Cell-Free Microbial DNA Using a Contaminant-Controlled Analysis Framework. Genome Biol..

[128] Orendain-Jaime E.N., Serafín-Higuera N., Leija-Montoya A.G., Martínez-Coronilla G., Moreno-Trujillo M., Sánchez-Muñoz F., Ruiz-Hernández A., González-Ramírez J. (2021). MicroRNAs Encoded by Virus and Small RNAs Encoded by Bacteria Associated with Oncogenic Processes. Processes.

[129] Zhan S., Wang Y., Chen X. (2020). RNA Virus-Encoded microRNAs: Biogenesis, Functions and Perspectives on Application. ExRNA.

[130] Diggins N.L., Hancock M.H. (2023). Viral miRNA Regulation of Host Gene Expression. Semin. Cell Dev. Biol..

[131] Pfeffer S., Zavolan M., Grässer F.A., Chien M., Russo J.J., Ju J., John B., Enright A.J., Marks D., Sander C. (2004). Identification of Virus-Encoded microRNAs. Science.

[132] Cai X., Schäfer A., Lu S., Bilello J.P., Desrosiers R.C., Edwards R., Raab-Traub N., Cullen B.R. (2006). Epstein-Barr Virus microRNAs Are Evolutionarily Conserved and Differentially Expressed. PLoS Pathog..

[133] Grundhoff A., Sullivan C.S., Ganem D. (2006). A Combined Computational and Microarray-Based Approach Identifies Novel microRNAs Encoded by Human Gamma-Herpesviruses. RNA.

[134] Lung R.W.-M., Tong J.H.-M., Sung Y.-M., Leung P.-S., Ng D.C.-H., Chau S.-L., Chan A.W.-H., Ng E.K.-O., Lo K.-W., To K.-F. (2009). Modulation of LMP2A Expression by a Newly Identified Epstein-Barr Virus-Encoded microRNA miR-BART22. Neoplasia.

[135] Zhang L., Yu J., Liu Z. (2020). MicroRNAs Expressed by Human Cytomegalovirus. Virol. J..

[136] Grey F., Antoniewicz A., Allen E., Saugstad J., McShea A., Carrington J.C., Nelson J. (2005). Identification and Characterization of Human Cytomegalovirus-Encoded microRNAs. J. Virol..

[137] Cui C., Griffiths A., Li G., Silva L.M., Kramer M.F., Gaasterland T., Wang X.-J., Coen D.M. (2006). Prediction and Identification of Herpes Simplex Virus 1-Encoded microRNAs. J. Virol..

[138] Umbach J.L., Kramer M.F., Jurak I., Karnowski H.W., Coen D.M., Cullen B.R. (2008). MicroRNAs Expressed by Herpes Simplex Virus 1 during Latent Infection Regulate Viral mRNAs. Nature.

[139] Jurak I., Kramer M.F., Mellor J.C., van Lint A.L., Roth F.P., Knipe D.M., Coen D.M. (2010). Numerous Conserved and Divergent microRNAs Expressed by Herpes Simplex Viruses 1 and 2. J. Virol..

[140] Umbach J.L., Wang K., Tang S., Krause P.R., Mont E.K., Cohen J.I., Cullen B.R. (2010). Identification of Viral microRNAs Expressed in Human Sacral Ganglia Latently Infected with Herpes Simplex Virus 2. J. Virol..

[141] Omoto S., Ito M., Tsutsumi Y., Ichikawa Y., Okuyama H., Brisibe E.A., Saksena N.K., Fujii Y.R. (2004). HIV-1 Nef Suppression by Virally Encoded microRNA. Retrovirology.

[142] Pfeffer S., Sewer A., Lagos-Quintana M., Sheridan R., Sander C., Grässer F.A., van Dyk L.F., Ho C.K., Shuman S., Chien M. (2005). Identification of microRNAs of the Herpesvirus Family. Nat. Methods.

[143] Ouellet D.L., Plante I., Landry P., Barat C., Janelle M.-E., Flamand L., Tremblay M.J., Provost P. (2008). Identification of Functional microRNAs Released through Asymmetrical Processing of HIV-1 TAR Element. Nucleic Acids Res..

[144] Duy J., Honko A.N., Altamura L.A., Bixler S.L., Wollen-Roberts S., Wauquier N., O’Hearn A., Mucker E.M., Johnson J.C., Shamblin J.D. (2018). Virus-Encoded miRNAs in Ebola Virus Disease. Sci. Rep..

[145] Seo G.J., Chen C.J., Sullivan C.S. (2009). Merkel Cell Polyomavirus Encodes a microRNA with the Ability to Autoregulate Viral Gene Expression. Virology.

[146] Meng Q., Sun H., Wu S., Familiari G., Relucenti M., Aschner M., Li X., Chen R. (2022). Epstein-Barr Virus-Encoded MicroRNA-BART18-3p Promotes Colorectal Cancer Progression by Targeting De Novo Lipogenesis. Adv. Sci..

[147] Cui C., Wang Y., Liu J., Zhao J., Sun P., Wang S. (2019). A Fungal Pathogen Deploys a Small Silencing RNA That Attenuates Mosquito Immunity and Facilitates Infection. Nat. Commun..

[148] Mathur M., Nair A., Kadoo N. (2020). Plant-Pathogen Interactions: MicroRNA-Mediated Trans-Kingdom Gene Regulation in Fungi and Their Host Plants. Genomics.

[149] Wong-Bajracharya J., Singan V.R., Monti R., Plett K.L., Ng V., Grigoriev I.V., Martin F.M., Anderson I.C., Plett J.M. (2022). The Ectomycorrhizal Fungus Encodes a microRNA Involved in Cross-Kingdom Gene Silencing during Symbiosis. Proc. Natl. Acad. Sci. USA.

[150] Lee H.-J., Hong S.-H. (2012). Analysis of microRNA-Size, Small RNAs in Streptococcus Mutans by Deep Sequencing. FEMS Microbiol. Lett..

[151] Diallo I., Provost P. (2020). RNA-Sequencing Analyses of Small Bacterial RNAs and Their Emergence as Virulence Factors in Host-Pathogen Interactions. Int. J. Mol. Sci..

[152] Gu H., Zhao C., Zhang T., Liang H., Wang X.-M., Pan Y., Chen X., Zhao Q., Li D., Liu F. (2017). Salmonella Produce microRNA-like RNA Fragment Sal-1 in the Infected Cells to Facilitate Intracellular Survival. Sci. Rep..

[153] Furuse Y., Finethy R., Saka H.A., Xet-Mull A.M., Sisk D.M., Smith K.L.J., Lee S., Coers J., Valdivia R.H., Tobin D.M. (2014). Search for microRNAs Expressed by Intracellular Bacterial Pathogens in Infected Mammalian Cells. PLoS ONE.

[154] Choi J.-W., Kim S.-C., Hong S.-H., Lee H.-J. (2017). Secretable Small RNAs via Outer Membrane Vesicles in Periodontal Pathogens. J. Dent. Res..

[155] Kreuzer-Redmer S., Bekurtz J.C., Arends D., Bortfeldt R., Kutz-Lohroff B., Sharbati S., Einspanier R., Brockmann G.A. (2016). Feeding of Enterococcus Faecium NCIMB 10415 Leads to Intestinal miRNA-423-5p-Induced Regulation of Immune-Relevant Genes. Appl. Environ. Microbiol..

[156] Zmora N., Suez J., Elinav E. (2019). You Are What You Eat: Diet, Health and the Gut Microbiota. Nat. Rev. Gastroenterol. Hepatol..

[157] Heydari Z., Rahaie M., Alizadeh A.M., Agah S., Khalighfard S., Bahmani S. (2019). Effects of Lactobacillus Acidophilus and Bifidobacterium Bifidum Probiotics on the Expression of MicroRNAs 135b, 26b, 18a and 155, and Their Involving Genes in Mice Colon Cancer. Probiotics Antimicrob. Proteins.

[158] Fahmy C.A., Gamal-Eldeen A.M., El-Hussieny E.A., Raafat B.M., Mehanna N.S., Talaat R.M., Shaaban M.T. (2019). Bifidobacterium Longum Suppresses Murine Colorectal Cancer through the Modulation of oncomiRs and Tumor Suppressor miRNAs. Nutr. Cancer.

[159] Singh R., Zogg H., Ro S. (2021). Role of microRNAs in Disorders of Gut-Brain Interactions: Clinical Insights and Therapeutic Alternatives. J. Pers. Med..

[160] Brennecke J., Stark A., Russell R.B., Cohen S.M. (2005). Principles of microRNA-Target Recognition. PLoS Biol..

[161] Baumann V., Winkler J. (2014). miRNA-Based Therapies: Strategies and Delivery Platforms for Oligonucleotide and Non-Oligonucleotide Agents. Future Med. Chem..

[162] Hofsli E., Sjursen W., Prestvik W.S., Johansen J., Rye M., Tranø G., Wasmuth H.H., Hatlevoll I., Thommesen L. (2013). Identification of Serum microRNA Profiles in Colon Cancer. Br. J. Cancer.

[163] Ghanbari R., Mosakhani N., Asadi J., Nouraee N., Mowla S.J., Poustchi H., Malekzadeh R., Knuutila S. (2015). Decreased Expression of Fecal miR-4478 and miR-1295b-3p in Early-Stage Colorectal Cancer. Cancer Biomark..

[164] Han H.-B., Gu J., Zuo H.-J., Chen Z.-G., Zhao W., Li M., Ji D.-B., Lu Y.-Y., Zhang Z.-Q. (2012). Let-7c Functions as a Metastasis Suppressor by Targeting MMP11 and PBX3 in Colorectal Cancer. J. Pathol..

[165] Silva C.M.S., Barros-Filho M.C., Wong D.V.T., Mello J.B.H., Nobre L.M.S., Wanderley C.W.S., Lucetti L.T., Muniz H.A., Paiva I.K.D., Kuasne H. (2021). Circulating Let-7e-5p, miR-106a-5p, miR-28-3p, and miR-542-5p as a Promising microRNA Signature for the Detection of Colorectal Cancer. Cancers.

[166] Dokhanchi M., Pakravan K., Zareian S., Hussen B.M., Farid M., Razmara E., Mossahebi-Mohammadi M., Cho W.C., Babashah S. (2021). Colorectal Cancer Cell-Derived Extracellular Vesicles Transfer miR-221-3p to Promote Endothelial Cell Angiogenesis via Targeting Suppressor of Cytokine Signaling 3. Life Sci..

[167] Cho W.-C., Kim M., Park J.W., Jeong S.-Y., Ku J.-L. (2021). Exosomal miR-193a and Let-7g Accelerate Cancer Progression on Primary Colorectal Cancer and Paired Peritoneal Metastatic Cancer. Transl. Oncol..

[168] Wang J., Huang S.-K., Zhao M., Yang M., Zhong J.-L., Gu Y.-Y., Peng H., Che Y.-Q., Huang C.-Z. (2014). Identification of a Circulating microRNA Signature for Colorectal Cancer Detection. PLoS ONE.

[169] Wang X., Gao G., Chen Z., Chen Z., Han M., Xie X., Jin Q., Du H., Cao Z., Zhang H. (2021). Identification of the miRNA Signature and Key Genes in Colorectal Cancer Lymph Node Metastasis. Cancer Cell Int..

[170] Zhang H., Zhu M., Shan X., Zhou X., Wang T., Zhang J., Tao J., Cheng W., Chen G., Li J. (2019). A Panel of Seven-miRNA Signature in Plasma as Potential Biomarker for Colorectal Cancer Diagnosis. Gene.

[171] Chen W.-Y., Zhao X.-J., Yu Z.-F., Hu F.-L., Liu Y.-P., Cui B.-B., Dong X.-S., Zhao Y.-S. (2015). The Potential of Plasma miRNAs for Diagnosis and Risk Estimation of Colorectal Cancer. Int. J. Clin. Exp. Pathol..

[172] Li J., Liu Y., Wang C., Deng T., Liang H., Wang Y., Huang D., Fan Q., Wang X., Ning T. (2015). Serum miRNA Expression Profile as a Prognostic Biomarker of Stage II/III Colorectal Adenocarcinoma. Sci. Rep..

[173] Luo X., Stock C., Burwinkel B., Brenner H. (2013). Identification and Evaluation of Plasma MicroRNAs for Early Detection of Colorectal Cancer. PLoS ONE.

[174] Ho G.Y.F., Jung H.J., Schoen R.E., Wang T., Lin J., Williams Z., Weissfeld J.L., Park J.Y., Loudig O., Suh Y. (2015). Differential Expression of Circulating microRNAs according to Severity of Colorectal Neoplasia. Transl. Res..

[175] Ahmed F.E., Amed N.C., Vos P.W., Bonnerup C., Atkins J.N., Casey M., Nuovo G.J., Naziri W., Wiley J.E., Allison R.R. (2012). Diagnostic microRNA Markers to Screen for Sporadic Human Colon Cancer in Blood. Cancer Genomics Proteomics.

[176] Liu D.-R., Guan Q.-L., Gao M.-T., Jiang L., Kang H.-X. (2016). miR-1260b Is a Potential Prognostic Biomarker in Colorectal Cancer. Med. Sci. Monit..

[177] Imaoka H., Toiyama Y., Fujikawa H., Hiro J., Saigusa S., Tanaka K., Inoue Y., Mohri Y., Mori T., Kato T. (2016). Circulating microRNA-1290 as a Novel Diagnostic and Prognostic Biomarker in Human Colorectal Cancer. Ann. Oncol..

[178] Liu X., Pan B., Sun L., Chen X., Zeng K., Hu X., Xu T., Xu M., Wang S. (2018). Circulating Exosomal miR-27a and miR-130a Act as Novel Diagnostic and Prognostic Biomarkers of Colorectal Cancer. Cancer Epidemiol. Biomarkers Prev..

[179] Xie B., Gong N., Guo Y., Hu G. (2021). MicroRNA-133b Expression Inversely Correlates with MET and Can Serve as an Optimum Predictive Biomarker for Patients of Colorectal Cancer. Transl. Cancer Res..

[180] Koga Y., Yasunaga M., Takahashi A., Kuroda J., Moriya Y., Akasu T., Fujita S., Yamamoto S., Baba H., Matsumura Y. (2010). MicroRNA Expression Profiling of Exfoliated Colonocytes Isolated from Feces for Colorectal Cancer Screening. Cancer Prev. Res..

[181] Uratani R., Toiyama Y., Kitajima T., Kawamura M., Hiro J., Kobayashi M., Tanaka K., Inoue Y., Mohri Y., Mori T. (2016). Diagnostic Potential of Cell-Free and Exosomal MicroRNAs in the Identification of Patients with High-Risk Colorectal Adenomas. PLoS ONE.

[182] Chen T., Cai S.-L., Li J., Qi Z.-P., Li X.-Q., Ye L.-C., Xie X.-F., Hou Y.-Y., Yao L.-Q., Xu M.-D. (2017). Mecp2-Mediated Epigenetic Silencing of miR-137 Contributes to Colorectal Adenoma-Carcinoma Sequence and Tumor Progression via Relieving the Suppression of c-Met. Sci. Rep..

[183] Ng L., Wan T.M.-H., Man J.H.-W., Chow A.K.-M., Iyer D., Chen G., Yau T.C.-C., Lo O.S.-H., Foo D.C.-C., Poon J.T.-C. (2017). Identification of Serum miR-139-3p as a Non-Invasive Biomarker for Colorectal Cancer. Oncotarget.

[184] Kanaan Z., Roberts H., Eichenberger M.R., Billeter A., Ocheretner G., Pan J., Rai S.N., Jorden J., Williford A., Galandiuk S. (2013). A Plasma microRNA Panel for Detection of Colorectal Adenomas: A Step toward More Precise Screening for Colorectal Cancer. Ann. Surg..

[185] Miyoshi J., Toden S., Yoshida K., Toiyama Y., Alberts S.R., Kusunoki M., Sinicrope F.A., Goel A. (2017). MiR-139-5p as a Novel Serum Biomarker for Recurrence and Metastasis in Colorectal Cancer. Sci. Rep..

[186] (2016). Systematic Analysis of Key miRNAs and Related Signaling Pathways in Colorectal Tumorigenesis. Gene.

[187] Li J.-M., Zhao R.-H., Li S.-T., Xie C.-X., Jiang H.-H., Ding W.-J., Du P., Chen W., Yang M., Cui L. (2012). Down-Regulation of Fecal miR-143 and miR-145 as Potential Markers for Colorectal Cancer. Saudi Med. J..

[188] Tsikitis V.L., Potter A., Mori M., Buckmeier J.A., Preece C.R., Harrington C.A., Bartley A.N., Bhattacharyya A.K., Hamilton S.R., Lance M.P. (2016). MicroRNA Signatures of Colonic Polyps on Screening and Histology. Cancer Prev. Res..

[189] Kalimutho M., Del Vecchio Blanco G., Di Cecilia S., Sileri P., Cretella M., Pallone F., Federici G., Bernardini S. (2011). Differential Expression of miR-144* as a Novel Fecal-Based Diagnostic Marker for Colorectal Cancer. J. Gastroenterol..

[190] Sun N., Zhang L., Zhang C., Yuan Y. (2020). miR-144-3p Inhibits Cell Proliferation of Colorectal Cancer Cells by Targeting BCL6 via Inhibition of Wnt/β-Catenin Signaling. Cell. Mol. Biol. Lett..

[191] Gao Z., Jiang J., Hou L., Zhang B. (2022). Dysregulation of MiR-144-5p/RNF187 Axis Contributes To the Progression of Colorectal Cancer. J. Transl. Int. Med..

[192] Ramzy I., Hasaballah M., Marzaban R., Shaker O., Soliman Z.A. (2015). Evaluation of microRNAs-29a, 92a and 145 in Colorectal Carcinoma as Candidate Diagnostic Markers: An Egyptian Pilot Study. Clin. Res. Hepatol. Gastroenterol..

[193] Xie Y., Zhang Y., Liu X., Cao L., Han M., Wang C., Chen J., Zhang X. (2023). miR-151a-5p Promotes the Proliferation and Metastasis of Colorectal Carcinoma Cells by Targeting AGMAT. Oncol. Rep..

[194] Lv Z.-C., Fan Y.-S., Chen H.-B., Zhao D.-W. (2015). Investigation of microRNA-155 as a Serum Diagnostic and Prognostic Biomarker for Colorectal Cancer. Tumour Biol..

[195] de Groen F.L.M., Timmer L.M., Menezes R.X., Diosdado B., Hooijberg E., Meijer G.A., Steenbergen R.D.M., Carvalho B. (2015). Oncogenic Role of miR-15a-3p in 13q Amplicon-Driven Colorectal Adenoma-to-Carcinoma Progression. PLoS ONE.

[196] Han L., Shi W.-J., Xie Y.-B., Zhang Z.-G. (2021). Diagnostic Value of Four Serum Exosome microRNAs Panel for the Detection of Colorectal Cancer. World J. Gastrointest. Oncol..

[197] Giráldez M.D., Lozano J.J., Ramírez G., Hijona E., Bujanda L., Castells A., Gironella M. (2013). Circulating microRNAs as Biomarkers of Colorectal Cancer: Results from a Genome-Wide Profiling and Validation Study. Clin. Gastroenterol. Hepatol..

[198] Fu F., Jiang W., Zhou L., Chen Z. (2018). Circulating Exosomal miR-17-5p and miR-92a-3p Predict Pathologic Stage and Grade of Colorectal Cancer. Transl. Oncol..

[199] Liu X., Xu T., Hu X., Chen X., Zeng K., Sun L., Wang S. (2018). Elevated Circulating miR-182 Acts as a Diagnostic Biomarker for Early Colorectal Cancer. Cancer Manag. Res..

[200] Perilli L., Vicentini C., Agostini M., Pizzini S., Pizzi M., D’Angelo E., Bortoluzzi S., Mandruzzato S., Mammano E., Rugge M. (2014). Circulating miR-182 Is a Biomarker of Colorectal Adenocarcinoma Progression. Oncotarget.

[201] Feng J., Wei Q., Yang M., Wang X., Liu B., Li J. (2021). Development and Validation of a Novel miRNA Classifier as a Prognostic Signature for Stage II/III Colorectal Cancer. Ann. Transl. Med..

[202] Yau T.O., Wu C.W., Dong Y., Tang C.-M., Ng S.S.M., Chan F.K.L., Sung J.J.Y., Yu J. (2014). microRNA-221 and microRNA-18a Identification in Stool as Potential Biomarkers for the Non-Invasive Diagnosis of Colorectal Carcinoma. Br. J. Cancer.

[203] Wikberg M.L., Myte R., Palmqvist R., van Guelpen B., Ljuslinder I. (2018). Plasma miRNA Can Detect Colorectal Cancer, but How Early?. Cancer Med..

[204] Zhang G.-J., Zhou T., Liu Z.-L., Tian H.-P., Xia S.-S. (2013). Plasma miR-200c and miR-18a as Potential Biomarkers for the Detection of Colorectal Carcinoma. Mol. Clin. Oncol..

[205] Basati G., Razavi A.E., Pakzad I., Malayeri F.A. (2016). Circulating Levels of the miRNAs, miR-194, and miR-29b, as Clinically Useful Biomarkers for Colorectal Cancer. Tumour Biol..

[206] Liu Z., Lu T., Wang Y., Jiao D., Li Z., Wang L., Liu L., Guo C., Zhao Y., Han X. (2021). Establishment and Experimental Validation of an Immune miRNA Signature for Assessing Prognosis and Immune Landscape of Patients with Colorectal Cancer. J. Cell. Mol. Med..

[207] Bilegsaikhan E., Liu H.N., Shen X.Z., Liu T.T. (2018). Circulating miR-338-5p Is a Potential Diagnostic Biomarker in Colorectal Cancer. J. Dig. Dis..

[208] Zheng G., Du L., Yang X., Zhang X., Wang L., Yang Y., Li J., Wang C. (2014). Serum microRNA Panel as Biomarkers for Early Diagnosis of Colorectal Adenocarcinoma. Br. J. Cancer.

[209] Ahmed F.E., Jeffries C.D., Vos P.W., Flake G., Nuovo G.J., Sinar D.R., Naziri W., Marcuard S.P. (2009). Diagnostic microRNA Markers for Screening Sporadic Human Colon Cancer and Active Ulcerative Colitis in Stool and Tissue. Cancer Genom. Proteom..

[210] Xu L., Li M., Wang M., Yan D., Feng G., An G. (2014). The Expression of microRNA-375 in Plasma and Tissue Is Matched in Human Colorectal Cancer. BMC Cancer.

[211] Chang P.-Y., Chen C.-C., Chang Y.-S., Tsai W.-S., You J.-F., Lin G.-P., Chen T.-W., Chen J.-S., Chan E.-C. (2016). MicroRNA-223 and microRNA-92a in Stool and Plasma Samples Act as Complementary Biomarkers to Increase Colorectal Cancer Detection. Oncotarget.

[212] Yang H., Lin J., Jiang J., Ji J., Wang C., Zhang J. (2020). miR-20b-5p Functions as Tumor Suppressor microRNA by Targeting cyclinD1 in Colon Cancer. Cell Cycle.

[213] Link A., Balaguer F., Shen Y., Nagasaka T., Lozano J.J., Boland C.R., Goel A. (2010). Fecal MicroRNAs as Novel Biomarkers for Colon Cancer Screening. Cancer Epidemiol. Biomarkers Prev..

[214] Du M., Liu S., Gu D., Wang Q., Zhu L., Kang M., Shi D., Chu H., Tong N., Chen J. (2014). Clinical Potential Role of Circulating microRNAs in Early Diagnosis of Colorectal Cancer Patients. Carcinogenesis.

[215] Nassar F.J., Msheik Z.S., Itani M.M., Helou R.E., Hadla R., Kreidieh F., Bejjany R., Mukherji D., Shamseddine A., Nasr R.R. (2021). Circulating miRNA as Biomarkers for Colorectal Cancer Diagnosis and Liver Metastasis. Diagnostics.

[216] Zanutto S., Pizzamiglio S., Ghilotti M., Bertan C., Ravagnani F., Perrone F., Leo E., Pilotti S., Verderio P., Gariboldi M. (2014). Circulating miR-378 in Plasma: A Reliable, Haemolysis-Independent Biomarker for Colorectal Cancer. Br. J. Cancer.

[217] Wang Q., Huang Z., Ni S., Xiao X., Xu Q., Wang L., Huang D., Tan C., Sheng W., Du X. (2012). Plasma miR-601 and miR-760 Are Novel Biomarkers for the Early Detection of Colorectal Cancer. PLoS ONE.

[218] Liu G.-H., Zhou Z.-G., Chen R., Wang M.-J., Zhou B., Li Y., Sun X.-F. (2013). Serum miR-21 and miR-92a as Biomarkers in the Diagnosis and Prognosis of Colorectal Cancer. Tumor Biology.

[219] Basati G., Emami Razavi A., Abdi S., Mirzaei A. (2014). Elevated Level of microRNA-21 in the Serum of Patients with Colorectal Cancer. Med. Oncol..

[220] Wu C.W., Ng S.S.M., Dong Y.J., Ng S.C., Leung W.W., Lee C.W., Wong Y.N., Chan F.K.L., Yu J., Sung J.J.Y. (2012). Detection of miR-92a and miR-21 in Stool Samples as Potential Screening Biomarkers for Colorectal Cancer and Polyps. Gut.

[221] Tsukamoto M., Iinuma H., Yagi T., Matsuda K., Hashiguchi Y. (2017). Circulating Exosomal MicroRNA-21 as a Biomarker in Each Tumor Stage of Colorectal Cancer. Oncology.

[222] Fukada M., Matsuhashi N., Takahashi T., Sugito N., Heishima K., Yoshida K., Akao Y. (2021). Postoperative Changes in Plasma miR21-5p as a Novel Biomarker for Colorectal Cancer Recurrence: A Prospective Study. Cancer Sci..

[223] Wang W., Qu A., Liu W., Liu Y., Zheng G., Du L., Zhang X., Yang Y., Wang C., Chen X. (2017). Circulating miR-210 as a Diagnostic and Prognostic Biomarker for Colorectal Cancer. Eur. J. Cancer Care.

[224] Yu H., Gao G., Jiang L., Guo L., Lin M., Jiao X., Jia W., Huang J. (2013). Decreased Expression of miR-218 Is Associated with Poor Prognosis in Patients with Colorectal Cancer. Int. J. Clin. Exp. Pathol..

[225] Pu X.-X., Huang G.-L., Guo H.-Q., Guo C.-C., Li H., Ye S., Ling S., Jiang L., Tian Y., Lin T.-Y. (2010). Circulating miR-221 Directly Amplified from Plasma Is a Potential Diagnostic and Prognostic Marker of Colorectal Cancer and Is Correlated with p53 Expression. J. Gastroenterol. Hepatol..

[226] Vychytilova-Faltejskova P., Radova L., Sachlova M., Kosarova Z., Slaba K., Fabian P., Grolich T., Prochazka V., Kala Z., Svoboda M. (2016). Serum-Based microRNA Signatures in Early Diagnosis and Prognosis Prediction of Colon Cancer. Carcinogenesis.

[227] Fang Z., Tang J., Bai Y., Lin H., You H., Jin H., Lin L., You P., Li J., Dai Z. (2015). Plasma Levels of microRNA-24, microRNA-320a, and microRNA-423-5p Are Potential Biomarkers for Colorectal Carcinoma. J. Exp. Clin. Cancer Res..

[228] Liang J., Tang J., Shi H., Li H., Zhen T., Duan J., Kang L., Zhang F., Dong Y., Han A. (2017). miR-27a-3p Targeting RXRα Promotes Colorectal Cancer Progression by Activating Wnt/β-Catenin Pathway. Oncotarget.

[229] Ostenfeld M.S., Jensen S.G., Jeppesen D.K., Christensen L.-L., Thorsen S.B., Stenvang J., Hvam M.L., Thomsen A., Mouritzen P., Rasmussen M.H. (2016). miRNA Profiling of Circulating EpCAM(+) Extracellular Vesicles: Promising Biomarkers of Colorectal Cancer. J. Extracell. Vesicles.

[230] Zhu Y., Xu A., Li J., Fu J., Wang G., Yang Y., Cui L., Sun J. (2016). Fecal miR-29a and miR-224 as the Noninvasive Biomarkers for Colorectal Cancer. Cancer Biomark..

[231] Huang Z., Huang D., Ni S., Peng Z., Sheng W., Du X. (2010). Plasma microRNAs Are Promising Novel Biomarkers for Early Detection of Colorectal Cancer. Int. J. Cancer.

[232] Tadano T., Kakuta Y., Hamada S., Shimodaira Y., Kuroha M., Kawakami Y., Kimura T., Shiga H., Endo K., Masamune A. (2016). MicroRNA-320 Family Is Downregulated in Colorectal Adenoma and Affects Tumor Proliferation by Targeting CDK6. World J. Gastrointest. Oncol..

[233] Liu X., Xu X., Pan B., He B., Chen X., Zeng K., Xu M., Pan Y., Sun H., Xu T. (2019). Circulating miR-1290 and miR-320d as Novel Diagnostic Biomarkers of Human Colorectal Cancer. J. Cancer.

[234] Cai R., Lu Q., Wang D. (2021). Construction and Prognostic Analysis of miRNA-mRNA Regulatory Network in Liver Metastasis from Colorectal Cancer. World J. Surg. Oncol..

[235] Song H., Ruan C., Xu Y., Xu T., Fan R., Jiang T., Cao M., Song J. (2022). Survival Stratification for Colorectal Cancer via Multi-Omics Integration Using an Autoencoder-Based Model. Exp. Biol. Med..

[236] Yu J., Jin L., Jiang L., Gao L., Zhou J., Hu Y., Li W., Zhi Q., Zhu X. (2016). Serum miR-372 Is a Diagnostic and Prognostic Biomarker in Patients with Early Colorectal Cancer. Anticancer Agents Med. Chem..

[237] Wang L., Song X., Yu M., Niu L., Zhao Y., Tang Y., Zheng B., Song X., Xie L. (2022). Serum Exosomal miR-377-3p and miR-381-3p as Diagnostic Biomarkers in Colorectal Cancer. Future Oncol..

[238] Wang S., Xiang J., Li Z., Lu S., Hu J., Gao X., Yu L., Wang L., Wang J., Wu Y. (2015). A Plasma microRNA Panel for Early Detection of Colorectal Cancer. Int. J. Cancer.

[239] Tan Y., Lin J.-J., Yang X., Gou D.-M., Fu L., Li F.-R., Yu X.-F. (2019). A Panel of Three Plasma microRNAs for Colorectal Cancer Diagnosis. Cancer Epidemiol..

[240] Krawczyk P., Powrózek T., Olesiński T., Dmitruk A., Dziwota J., Kowalski D., Milanowski J. (2017). Evaluation of miR-506 and miR-4316 Expression in Early and Non-Invasive Diagnosis of Colorectal Cancer. Int. J. Colorectal Dis..

[241] Lan S.-H., Lin S.-C., Wang W.-C., Yang Y.-C., Lee J.-C., Lin P.-W., Chu M.-L., Lan K.-Y., Zuchini R., Liu H.-S. (2021). Autophagy Upregulates miR-449a Expression to Suppress Progression of Colorectal Cancer. Front. Oncol..

[242] Zhang Z., Zhang D., Cui Y., Qiu Y., Miao C., Lu X. (2020). Identification of microRNA-451a as a Novel Circulating Biomarker for Colorectal Cancer Diagnosis. BioMed Res. Int..

[243] Yang I.-P., Tsai H.-L., Hou M.-F., Chen K.-C., Tsai P.-C., Huang S.-W., Chou W.-W., Wang J.-Y., Juo S.-H.H. (2012). MicroRNA-93 Inhibits Tumor Growth and Early Relapse of Human Colorectal Cancer by Affecting Genes Involved in the Cell Cycle. Carcinogenesis.

[244] Sun Y., Liu Y., Cogdell D., Calin G.A., Sun B., Kopetz S., Hamilton S.R., Zhang W. (2016). Examining Plasma microRNA Markers for Colorectal Cancer at Different Stages. Oncotarget.

[245] Chen Y., Liu H., Ning S., Wei C., Li J., Wei W., Zhang L. (2022). The High Ratio of the Plasma miR-96/miR-99b Correlated With Poor Prognosis in Patients With Metastatic Colorectal Cancer. Front. Mol. Biosci..

[246] Hibino Y., Sakamoto N., Naito Y., Goto K., Oo H.Z., Sentani K., Hinoi T., Ohdan H., Oue N., Yasui W. (2015). Significance of miR-148a in Colorectal Neoplasia: Downregulation of miR-148a Contributes to the Carcinogenesis and Cell Invasion of Colorectal Cancer. Pathobiology.

