# Supplementary material for: Cross-Kingdom Interaction of miRNAs and Gut Microbiota with Non-Invasive Diagnostic and Therapeutic Implications in Colorectal Cancer
Source: Int J Mol Sci. 2023 Jun 23;24(13):10520. doi: 10.3390/ijms241310520 (PMC10341587; doi:10.3390/ijms241310520)
Supplement: Supplementary file 1 [file ijms-24-10520-s001.zip › ijms-2429038 - Supplementary Table S1.pdf]

miRNA	Status in CRC	miRNA Source:	Reference	Ref. No.
led 7d	Downregulated	serum	(Hofsli et al. 2013)	[162]
let-7a-5p	Downregulated	stool	(Ghanbari et al. 2015)	[163]
let-7c	Downregulated	cell culture	(Han et al. 2012)	[164]
let-7e-5p	Upregulated	plasma	(Silva et al. 2021)	[165]
let-7f-5p	Downregulated	stool	(Ghanbari et al. 2015)	[163]
let-7f-5p	Upregulated	serum	(Dokhanchi et al. 2021)	[166]
let-7g	Upregulated	plasma	(Cho et al. 2021)	[167]
let-7g	Upregulated	serum	(Wang et al. 2014)	[168]
miR-100	Downregulated	lymph node tissue	(Wang et al. 2021)	[169]
miR-103	Downregulated	serum	(Hofsli et al. 2013)	[162]
miR-103a-3p	Upregulated	cell culture	(Zhang et al. 2019)	[170]
miR-106a	Upregulated	plasma	(Silva et al. 2021)	[165]
miR-106a	Downregulated	serum	(Hofsli et al. 2013)	[162]
miR-106a	Upregulated	plasma	(Chen et al. 2015)	[171]
miR-106a	Upregulated	stool	(Koga et al. 2013)	[49]
miR-106a	Upregulated	serum	(Li et al. 2015)	[172]
miR-106a-5p	Upregulated	plasma	(Silva et al. 2021)	[165]
miR-106b	Upregulated	plasma	(Luo et al. 2013)	[173]
miR-107	Downregulated	serum	(Hofsli et al. 2013)	[162]
miR-1180	Upregulated	serum	(Ho et al. 2015)	[174]
miR-124	Downregulated	plasma	(Ahmed et al. 2012)	[175]
miR-125	Upregulated	blood	(B. Chen et al. 2019)	[48]
miR-1260b	Upregulated	tumor tissue	(Liu et al. 2016)	[176]
miR-127-3p	Upregulated	plasma	(Zhang et al. 2019)	[170]
miR-127-5p	Downregulated	plasma	(Ahmed et al. 2012)	[175]
miR-1290	Upregulated	serum	(Imaoka et al. 2016)	[177]
miR-1295b-4p	Downregulated	stool	(Ghanbari et al. 2015)	[163]
miR-130a	Upregulated	plasma	(X. Liu, Pan, et al. 2018)	[178]
miR-133a	Upregulated	plasma	(Luo et al. 2013)	[173]
miR-133b	Downregulated	tumor tissue	(Xie et al. 2021)	[179]
miR-135b	Upregulated	stool	(Wu et al. 2014)	[27]
miR-135b	Upregulated	stool	(Koga et al. 2010)	[180]
miR-135b	Upregulated	tumor tissue	(Uratani et al. 2016)	[181]
miR-137	Downregulated	tumor tissue	(Chen et al. 2017)	[182]
miR-138	Downregulated	plasma	(Ahmed et al. 2012)	[175]
miR-139-3p	Downregulated	serum	(L. Ng et al. 2017)	[183]
miR-139-p3	Upregulated	plasma	(Kanaan et al. 2013)	[184]
miR-139-5p	Upregulated	serum	(Miyoshi et al. 2017)	[185]
miR-142-3p	Downregulated	plasma	(Ghanbari et al. 2015)	[163]
miR-142-5p	Upregulated	tissue	(Yin 2016)	[186]
miR-143	Downregulated	serum	(Hofsli et al. 2013)	[162]
miR-143	Downregulated	stool	(Li et al. 2012)	[187]
miR-143	Downregulated	tumor tissue	(Tsikitis et al. 2016)	[188]
miR-143	Downregulated	plasma	(Ahmed et al. 2012)	[175]
miR-143	Upregulated	plasma	(Luo et al. 2013)	[173]

miR-144	Upregulated	stool	(Kalimutho et al. 2011)	[189]
miR-144-3p	Downregulated	tumor tissue	(Sun et al. 2020)	[190]
miR-144-5p	Downregulated	tumor tissue	(Gao et al. 2022)	[191]
miR-145	Downregulated	serum	(Li et al. 2015)	[172]
miR-145	Downregulated	tumor tissue	(Tsikitis et al. 2016)	[188]
miR-145	Downregulated	serum	(Ramzy et al. 2015)	[192]
miR-145	Downregulated	stool	(Li et al. 2012)	[187]
miR-145	Downregulated	tumor tissue	(Yin 2016)	[186]
miR-145	Upregulated	plasma	(Luo et al. 2013)	[173]
miR-146a	Downregulated	plasma	(Ahmed et al. 2012)	[175]
miR-151-5p	Downregulated	serum	(Hofsli et al. 2013)	[162]
miR-151a-5p	Upregulated	cell lines	(Xie et al. 2023)	[193]
miR-155	Upregulated	serum	(Lv et al. 2015)	[194]
miR-155	Upregulated	serum	(Hofsli et al. 2013)	[162]
miR-15a-3p	Upregulated	tumor tissue	(de Groen et al. 2015)	[195]
miR-15b	Upregulated	serum	(Han et al. 2021)	[196]
miR-15b	Upregulated	plasma	(Giráldez et al. 2013)	[197]
miR-15b	Upregulated	plasma	(Herreros-Villanueva et al. 2019)	[43]
miR-16	Upregulated	serum	(Han et al. 2021)	[196]
miR-17	Upregulated	tumor tissue	(de Groen et al. 2015)	[195]
miR-17-3p	Upregulated	plasma	(Ng et al. 2009)	[19]
miR-17-3p	Upregulated	serum	(Li et al. 2015)	[172]
miR-17-3p	Upregulated	plasma	(Ahmed et al. 2012)	[175]
miR-17-5p	Upregulated	serum	(Fu et al. 2018)	[198]
miR-17-92	Upregulated	stool	(Koga et al. 2010)	[180]
miR-181a-3p	Upregulated	plasma	(Zhang et al. 2019)	[170]
miR-181b	Downregulated	serum	(Wang et al. 2014)	[168]
miR-182	Upregulated	plasma	(X. Liu, Xu, et al. 2018)	[199]
miR-182	Upregulated	plasma	(Perilli et al. 2014)	[200]
miR-183	Upregulated	tumor tisssue	(Feng et al. 2021)	[201]
miR-18a	Upregulated	stool	(Yau et al. 2014)	[202]
miR-18a	Upregulated	plasma	(Giráldez et al. 2013)	[197]
miR-18a	Upregulated	plasma	(Luo et al. 2013)	[173]
miR-18a	Upregulated	plasma	(Wikberg et al. 2018)	[203]
miR-18a	Upregulated	plasma	(Herreros-Villanueva et al. 2019)	[43]
miR-18a	Upregulated	tumor tissue, plasma	(Zhang et al. 2013)	[204]
miR-18a	Upregulated	tumor tisssue	(Yin 2016)	[186]
miR-18a-5p	Upregulated	plasma	(Zhang et al. 2019)	[170]
miR-18b	Upregulated	tumor tissue	(Yin 2016)	[186]
miR-18b-5p	Upregulated	plasma	(Zhang et al. 2019)	[170]
miR-191	Downregulated	serum	(Hofsli et al. 2013)	[162]
miR-193a	Downregulated	plasma	(Cho et al. 2021)	[167]
miR-194	Downregulated	serum	(Basati et al. 2016)	[205]
miR-194-3p	Downregulated	tumor tissue	(Liu et al. 2021)	[206]
miR-195	Downregulated	tumor tissue	(Feng et al. 2021)	[201]
miR-196a	Upregulated	plasma	(Ahmed et al. 2012)	[175]

miR-196b	Upregulated	tumor tissue	(Bilegsaikhan et al. 2018)	[207]
miR-199-3p	Downregulated	serum	(Hofsli et al. 2013)	[162]
miR-19a	Upregulated	plasma	(Giráldez et al. 2013)	[197]
miR-19a-3p	Upregulated	serum	(Zheng et al. 2014)	[208]
miR-19a	Upregulated	plasma	(Herreros-Villanueva et al. 2019)	[43]
miR-19a-3p	Upregulated	serum	(Zheng et al. 2014)	[208]
miR-19a-3p	Upregulated	serum	(Feng et al. 2021)	[201]
miR-19b	Upregulated	plasma	(Giráldez et al. 2013)	[197]
miR-19b	Upregulated	plasma	(Herreros-Villanueva et al. 2019)	[43]
miR-200c	Upregulated	plasma	(Zhang et al. 2013)	[204]
miR-203-3p	Upregulated	serum	(Dokhanchi et al. 2021)	[166]
miR-203	Downregulated	serum	(Wang et al. 2014)	[168]
miR-203	Upregulated	stool	(Ahmed et al. 2009)	[209]
miR-203-3p	Upregulated	serum	(Dokhanchi et al. 2021)	[166]
miR-206	Upregulated and Downregulated*	plasma, tumor tissue	(Xu et al. 2014)	[210]
miR-20a	Upregulated	stool	(Ahmed et al. 2009)	[209]
miR-20a	Upregulated	stool	(Chang et al. 2016)	[211]
miR-20a	Upregulated	plasma	(Chen et al. 2015)	[171]
miR-20a	Upregulated	plasma	(Ahmed et al. 2012)	[175]
miR-20a	Upregulated	plasma	(Luo et al. 2013)	[173]
miR-20a	Upregulated	tumor tissue	(Feng et al. 2021)	[201]
miR-20a	Upregulated	tumor tissue	(de Groen et al. 2015)	[195]
miR-20b-5p	Downregulated	tumor tissue	(Yang et al. 2020)	[212]
miR-21	Upregulated	stool	(Link et al. 2010)	[213]
miR-21	Upregulated	plasma	(Luo et al. 2013)	[173]
miR-21	Upregulated	plasma	(Wikberg et al. 2018)	[203]
miR-21	Upregulated	plasma	(Du et al. 2014)	[214]
miR-21	Upregulated	plasma	(Nassar et al. 2021)	[215]
miR-21	Upregulated	plasma	(Ahmed et al. 2012)	[175]
miR-21	Upregulated	plasma	(Zanutto et al. 2014)	[216]
miR-21	Upregulated	plasma	(Kanaan et al. 2013)	[184]
miR-21	Upregulated	serum	(Wang et al. 2012)	[217]
miR-21	Upregulated	serum	(Liu et al. 2013)	[218]
miR-21	Upregulated	serum	(Toiyama et al. 2013)	[26]
miR-21	Upregulated	serum	(Basati et al. 2014)	[219]
miR-21	Upregulated	stool	(Wu et al. 2012)	[220]
miR-21	Upregulated	serum	(Han et al. 2021)	[196]
miR-21	Upregulated	tumor tissue	(Feng et al. 2021)	[201]
miR-21	Upregulated	serum	(Tsukamoto et al. 2017)	[221]
miR-21	Upregulated	serum, exosomal	(Uratani et al. 2016)	[181]
miR-21-5p	Upregulated	plasma	(Fukada et al. 2021)	[222]
miR-210	Upregulated	serum	(Wang et al. 2017)	[223]
miR-210	Upregulated	serum	(Hofsli et al. 2013)	[162]
miR-210	Upregulated	plasma	(Nassar et al. 2021)	[215]
miR-212	Upregulated	tumor tissue	Yin/2016 [69]	[186]

miR-214	Upregulated	plasma	(Ahmed et al. 2012)	[175]
miR-216a-5p	Upregulated	tumor tissue	(Liu et al. 2021)	[206]
miR-218	Downregulated	tumor tissue, serum	(Yu et al. 2013)	[224]
miR-22	Upregulated	plasma	(Wikberg et al. 2018)	[203]
miR-221	Downregulated	serum	(Hofsli et al. 2013)	[162]
miR-221	Upregulated	plasma	(Pu et al. 2010)	[225]
miR-221	Upregulated	stool	(Yau et al. 2014)	[202]
miR-221-3p	Upregulated	serum	(Dokhanchi et al. 2021)	[166]
miR-222	*Inconclusive*	plasma	(Ahmed et al. 2012)	[175]
miR-223	Upregulated	serum	(Tsukamoto et al. 2017)	[221]
miR-223	Upregulated	stool	(Phua et al. 2014)	[20]
miR-223	Upregulated	stool, plasma	(Chang et al. 2016)	[211]
miR-23a-3p	Upregulated	serum	(Vychytilova-Faltejskova et al. 2016)	[226]
miR-24	Downregulated	plasma	(Fang et al. 2015)	[227]
miR-25	Upregulated	plasma	(Wikberg et al. 2018)	[203]
miR-25	Upregulated	serum	(Ho et al. 2015)	[174]
miR-26a-5p	Downregulated	plasma	(Ghanbari et al. 2015)	[163]
miR-27a	Upregulated	plasma	(X. Liu, Pan, et al. 2018)	[178]
miR-27a-3p	Upregulated	tumor tissue	(Liang et al. 2017)	[228]
miR-27a-3p	Upregulated	tumor tissue, plasma	(Ostenfeld et al. 2016)	[229]
miR-27a-3p	Upregulated	serum	(Vychytilova-Faltejskova et al. 2016)	[226]
miR-28-3p	Upregulated	plasma	(Silva et al. 2021)	[165]
miR-29a	Upregulated and Downregulated**	stool	(Zhu et al. 2016)	[230]
miR-29a	Upregulated	plasma	(Wang et al. 2012)	[217]
miR-29a	Upregulated	plasma	(Giráldez et al. 2013)	[197]
miR-29a	Upregulated	plasma	(Luo et al. 2013)	[173]
miR-29a	Upregulated	plasma	(Herreros-Villanueva et al. 2019)	[43]
miR-29a	Upregulated	plasma	(Huang et al. 2010)	[231]
miR-29a	Upregulated	serum	(Hofsli et al. 2013)	[162]
miR-29b	Downregulated	serum	(Basati et al. 2016)	[205]
miR-30b	Downregulated	serum	(Ho et al. 2015)	[174]
miR-30a	Downregulated	tumor tissue	(Tsikitis et al. 2016)	[188]
miR-30b	Downregulated	serum	(Ho et al. 2015)	[174]
miR-31	Upregulated	tumor tissue	(Wu et al. 2014)	[27]
miR-31	Upregulated	tumor tissue	(Yin 2016)	[186]
miR-31	Upregulated	serum	(Han et al. 2021)	[196]
miR-31	Downregulated	serum	(Wang et al. 2014)	[168]
miR-320	Downregulated	tumor tissue	(Tadano et al. 2016)	[232]
miR-320a	Downregulated	plasma	(Fang et al. 2015)	[227]
miR-320a	Upregulated	serum	(Hofsli et al. 2013)	[162]
miR-320d	Upregulated and Downregulated*	tumor tissue, plasma	(X. Liu et al. 2019)	[233]
miR-335	Upregulated	plasma	(Giráldez et al. 2013)	[197]
miR-335	Upregulated	plasma	(Herreros-Villanueva et al. 2019)	[43]

miR-338-5p	Upregulated	serum	(Bilegsaikhan et al. 2018)	[207]
miR-33b-5p	Downregulated	tumor tissue	(Cai et al. 2021)	[234]
miR-34c	Upregulated	tumor tissue	(Song et al. 2022)	[235]
miR-3677-3p	Downregulated	tumor tissue	(Liu et al. 2021)	[206]
miR-372	Upregulated	serum	(Yu et al. 2016)	[236]
miR-375	Downregulated	tumor tissue, plasma	(Xu et al. 2014)	[210]
miR-376c-3p	Upregulated	serum	(Vychytilova-Faltejskova et al. 2016)	[226]
miR-377-3p	Downregulated	serum	(Wang et al. 2022)	[237]
miR-378	Upregulated	plasma	(Zanutto et al. 2014)	[216]
miR-378	Upregulated	serum	(Hofsli et al. 2013)	[162]
miR-381-3p	Downregulated	serum	(Wang et al. 2022)	[237]
miR-382	Downregulated	serum	(Hofsli et al. 2013)	[162]
miR-409-3p	Downregulated	serum	(Hofsli et al. 2013)	[162]
miR-409-3p	Upregulated	plasma	(Wang et al. 2015)	[238]
miR-422a	Downregulated	serum	(Zheng et al. 2014)	[208]
miR-423-3p	Downregulated	serum	(Hofsli et al. 2013)	[162]
miR-423-5p	Downregulated	plasma	(Fang et al. 2015)	[227]
miR-423-5p	Upregulated	serum	(Hofsli et al. 2013)	[162]
miR-425-5p	Upregulated	plasma	(Tan et al. 2019)	[239]
miR-431	Upregulated	plasma	(Kanaan et al. 2013)	[184]
miR-4316	Upregulated	plasma	(Krawczyk et al. 2017)	[240]
miR-4478	Downregulated	stool	(Ghanbari et al. 2015)	[163]
miR-449a	Downregulated	tumor tissue	(Lan et al. 2021)	[241]
miR-451	Downregulated	tumor tissue	Yin/2016 [69]	[186]
miR-451	Upregulated	stool	(Phua et al. 2014)	[20]
miR-451a	Downregulated	serum	(Zhang et al. 2020)	[242]
miR-486	Upregulated	serum	(Ho et al. 2015)	[174]
miR-506	Upregulated	plasma	(Krawczyk et al. 2017)	[240]
miR-542-5p	Upregulated	plasma	(Silva et al. 2021)	[165]
miR-601	Downregulated	plasma	(Wang et al. 2012)	[217]
miR-638	Downregulated	tumor tissue	(Yin 2016)	[186]
miR-652	Downregulated	serum	(Hofsli et al. 2013)	[162]
miR-7	Upregulated	plasma	(Ahmed et al. 2012)	[175]
miR-7	Upregulated and Downregulated	plasma	(Wang et al. 2012)	[238]
miR-720	Upregulated	serum	(Hofsli et al. 2013)	[162]
miR-760	Downregulated	plasma	(Wang et al. 2012)	[217]
miR-92a-3p	Upregulated	serum	(Fu et al. 2018)	[198]
miR-92	Upregulated	plasma	Ng et al, 2009	[19]
miR-92a	Upregulated	serum	(Liu et al. 2013)	[218]
miR-92a	Upregulated	serum	(Uratani et al. 2016)	[181]
miR-92a	Upregulated	serum	(Hofsli et al. 2013)	[162]
miR-92a	Upregulated	plasma	(Ahmed et al. 2012)	[175]
miR-92a	Upregulated	plasma	(Huang et al. 2010)	[231]
miR-92a	Upregulated	plasma	(Wang et al. 2012)	[217]
miR-92a	Upregulated	plasma	(Luo et al. 2013)	[173]

miR-92a	Upregulated	stool, plasma	(Chang et al. 2016)	[211]
miR-92a	Upregulated	stool	(Wu et al. 2012)	[220]
miR-92a-3p	Upregulated	serum	(Zheng et al. 2014)	[208]
miR-93	*Inconclusive*	tumor tissue	(Yang et al. 2012)	[243]
miR-96	Upregulated	plasma	(Ahmed et al. 2012)	[175]
miR-96	Upregulated	plasma	(Sun et al. 2016)	[244]
miR-96	Upregulated	plasma	(Chen et al. 2022)	[245]
miR-99a	Downregulated	lymph node tissue	(Wang et al. 2021)	[169]
miR-99b	Downregulated	plasma	(Chen et al. 2022)	[245]
mi-148a	Downregulated	tumor tissue	(Hibino et al. 2015)	[246]
miRNA-139	Downregulated	tumor tissue	(Feng et al. 2021)	[201]

*** If differential expression of certain miRNA appears in various tissues, type of tissue follows the same order as type of miRNA expression**

**** Upregulated in rectal carcinoma, downregulated in colon carcinoma**

References

162. Hofsli, E.; Sjursen, W.; Prestvik, W.S.; Johansen, J.; Rye, M.; Tranø, G.; Wasmuth, H.H.; Hatlevoll, I.; Thommesen, L. Identification of Serum microRNA Profiles in Colon Cancer. *Br. J. Cancer* **2013**, *108*, 1712–1719.
163. Ghanbari, R.; Mosakhani, N.; Asadi, J.; Nouraee, N.; Mowla, S.J.; Poustchi, H.; Malekzadeh, R.; Knuutila, S. Decreased Expression of Fecal miR-4478 and miR-1295b-3p in Early-Stage Colorectal Cancer. *Cancer Biomark.* **2015**, *15*, 189–195.
164. Han, H.-B.; Gu, J.; Zuo, H.-J.; Chen, Z.-G.; Zhao, W.; Li, M.; Ji, D.-B.; Lu, Y.-Y.; Zhang, Z.-Q. Let-7c Functions as a Metastasis Suppressor by Targeting MMP11 and PBX3 in Colorectal Cancer. *J. Pathol.* **2012**, *226*, 544–555.
165. Silva, C.M.S.; Barros-Filho, M.C.; Wong, D.V.T.; Mello, J.B.H.; Nobre, L.M.S.; Wanderley, C.W.S.; Lucetti, L.T.; Muniz, H.A.; Paiva, I.K.D.; Kuasne, H.; et al. Circulating Let-7e-5p, miR-106a-5p, miR-28-3p, and miR-542-5p as a Promising microRNA Signature for the Detection of Colorectal Cancer. *Cancers* **2021**, *13*, 1493.
166. Dokhanchi, M.; Pakravan, K.; Zareian, S.; Hussen, B.M.; Farid, M.; Razmara, E.; Mossahebi-Mohammadi, M.; Cho, W.C.; Babashah, S. Colorectal Cancer Cell-Derived Extracellular Vesicles Transfer miR-221-3p to Promote Endothelial Cell Angiogenesis via Targeting Suppressor of Cytokine Signaling 3. *Life Sci.* **2021**, *285*, 119937.
167. Cho, W.-C.; Kim, M.; Park, J.W.; Jeong, S.-Y.; Ku, J.-L. Exosomal miR-193a and Let-7g Accelerate Cancer Progression on Primary Colorectal Cancer and Paired Peritoneal Metastatic Cancer. *Transl. Oncol.* **2021**, *14*, 101000.
168. Wang, J.; Huang, S.-K.; Zhao, M.; Yang, M.; Zhong, J.-L.; Gu, Y.-Y.; Peng, H.; Che, Y.-Q.; Huang, C.-Z. Identification of a Circulating microRNA Signature for Colorectal Cancer Detection. *PLoS One* **2014**, *9*, e87451.
169. Wang, X.; Gao, G.; Chen, Z.; Chen, Z.; Han, M.; Xie, X.; Jin, Q.; Du, H.; Cao, Z.; Zhang, H. Identification of the miRNA Signature and Key Genes in Colorectal Cancer Lymph Node Metastasis. *Cancer Cell Int.* **2021**, *21*, 1–12.
170. Zhang, H.; Zhu, M.; Shan, X.; Zhou, X.; Wang, T.; Zhang, J.; Tao, J.; Cheng, W.; Chen, G.; Li, J.; et al. A Panel of Seven-miRNA Signature in Plasma as Potential Biomarker for Colorectal Cancer Diagnosis. *Gene* **2019**, *687*, 246–254.
171. Chen, W.-Y.; Zhao, X.-J.; Yu, Z.-F.; Hu, F.-L.; Liu, Y.-P.; Cui, B.-B.; Dong, X.-S.; Zhao, Y.-S. The Potential of Plasma miRNAs for Diagnosis and Risk Estimation of Colorectal Cancer. *Int. J. Clin. Exp. Pathol.* **2015**, *8*, 7092–7101.
172. Li, J.; Liu, Y.; Wang, C.; Deng, T.; Liang, H.; Wang, Y.; Huang, D.; Fan, Q.; Wang, X.; Ning, T.; et al. Serum miRNA Expression Profile as a Prognostic Biomarker of Stage II/III Colorectal Adenocarcinoma. *Sci. Rep.* **2015**, *5*, 12921.
173. Luo, X.; Stock, C.; Burwinkel, B.; Brenner, H. Identification and Evaluation of Plasma MicroRNAs for Early Detection of Colorectal Cancer. *PLoS One* **2013**, *8*, e62880.
174. Ho, G.Y.F.; Jung, H.J.; Schoen, R.E.; Wang, T.; Lin, J.; Williams, Z.; Weissfeld, J.L.; Park, J.Y.; Loudig, O.; Suh, Y. Differential Expression of Circulating microRNAs according to Severity of Colorectal Neoplasia. *Transl. Res.* **2015**, *166*, 225–232.
175. Ahmed, F.E.; Amed, N.C.; Vos, P.W.; Bonnerup, C.; Atkins, J.N.; Casey, M.; Nuovo, G.J.; Naziri, W.; Wiley, J.E.; Allison, R.R. Diagnostic microRNA Markers to Screen for Sporadic Human Colon Cancer in Blood. *Cancer Genomics Proteomics* **2012**, *9*, 179–192.
176. Liu, D.-R.; Guan, Q.-L.; Gao, M.-T.; Jiang, L.; Kang, H.-X. miR-1260b Is a Potential Prognostic Biomarker in Colorectal Cancer. *Med. Sci. Monit.* **2016**, *22*, 2417–2423.
177. Imaoka, H.; Toiyama, Y.; Fujikawa, H.; Hiro, J.; Saigusa, S.; Tanaka, K.; Inoue, Y.; Mohri, Y.; Mori, T.; Kato, T.; et al. Circulating microRNA-1290 as a Novel Diagnostic and Prognostic Biomarker in Human Colorectal Cancer. *Ann. Oncol.* **2016**, *27*, 1879–1886.
178. Liu, X.; Pan, B.; Sun, L.; Chen, X.; Zeng, K.; Hu, X.; Xu, T.; Xu, M.; Wang, S. Circulating Exosomal miR-27a and miR-130a Act as Novel Diagnostic and Prognostic Biomarkers of Colorectal Cancer. *Cancer Epidemiol. Biomarkers Prev.* **2018**, *27*, 746–754.

179. Xie, B.; Gong, N.; Guo, Y.; Hu, G. MicroRNA-133b Expression Inversely Correlates with MET and Can Serve as an Optimum Predictive Biomarker for Patients of Colorectal Cancer. *Transl. Cancer Res.* **2021**, *10*, 57–64.
180. Koga, Y.; Yasunaga, M.; Takahashi, A.; Kuroda, J.; Moriya, Y.; Akasu, T.; Fujita, S.; Yamamoto, S.; Baba, H.; Matsumura, Y. MicroRNA Expression Profiling of Exfoliated Colonocytes Isolated from Feces for Colorectal Cancer Screening. *Cancer Prev. Res.* **2010**, *3*, 1435–1442.
181. Uratani, R.; Toiyama, Y.; Kitajima, T.; Kawamura, M.; Hiro, J.; Kobayashi, M.; Tanaka, K.; Inoue, Y.; Mohri, Y.; Mori, T.; et al. Diagnostic Potential of Cell-Free and Exosomal MicroRNAs in the Identification of Patients with High-Risk Colorectal Adenomas. *PLoS One* **2016**, *11*, e0160722.
182. Chen, T.; Cai, S.-L.; Li, J.; Qi, Z.-P.; Li, X.-Q.; Ye, L.-C.; Xie, X.-F.; Hou, Y.-Y.; Yao, L.-Q.; Xu, M.-D.; et al. Mecp2-Mediated Epigenetic Silencing of miR-137 Contributes to Colorectal Adenoma-Carcinoma Sequence and Tumor Progression via Relieving the Suppression of c-Met. *Sci. Rep.* **2017**, *7*, 44543.
183. Ng, L.; Wan, T.M.-H.; Man, J.H.-W.; Chow, A.K.-M.; Iyer, D.; Chen, G.; Yau, T.C.-C.; Lo, O.S.-H.; Foo, D.C.-C.; Poon, J.T.-C.; et al. Identification of Serum miR-139-3p as a Non-Invasive Biomarker for Colorectal Cancer. *Oncotarget* **2017**, *8*, 27393–27400.
184. Kanaan, Z.; Roberts, H.; Eichenberger, M.R.; Billeter, A.; Ocheretner, G.; Pan, J.; Rai, S.N.; Jorden, J.; Williford, A.; Galandiuk, S. A Plasma microRNA Panel for Detection of Colorectal Adenomas: A Step toward More Precise Screening for Colorectal Cancer. *Ann. Surg.* **2013**, *258*, 400–408.
185. Miyoshi, J.; Toden, S.; Yoshida, K.; Toiyama, Y.; Alberts, S.R.; Kusunoki, M.; Sinicrope, F.A.; Goel, A. MiR-139-5p as a Novel Serum Biomarker for Recurrence and Metastasis in Colorectal Cancer. *Sci. Rep.* **2017**, *7*, 43393.
186. Systematic Analysis of Key miRNAs and Related Signaling Pathways in Colorectal Tumorigenesis. *Gene* **2016**, *578*, 177–184.
187. Li, J.-M.; Zhao, R.-H.; Li, S.-T.; Xie, C.-X.; Jiang, H.-H.; Ding, W.-J.; Du, P.; Chen, W.; Yang, M.; Cui, L. Down-Regulation of Fecal miR-143 and miR-145 as Potential Markers for Colorectal Cancer. *Saudi Med. J.* **2012**, *33*, 24–29.
188. Tsikitis, V.L.; Potter, A.; Mori, M.; Buckmeier, J.A.; Preece, C.R.; Harrington, C.A.; Bartley, A.N.; Bhattacharyya, A.K.; Hamilton, S.R.; Lance, M.P.; et al. MicroRNA Signatures of Colonic Polyps on Screening and Histology. *Cancer Prev. Res.* **2016**, *9*, 942–949.
189. Kalimutho, M.; Del Vecchio Blanco, G.; Di Cecilia, S.; Sileri, P.; Cretella, M.; Pallone, F.; Federici, G.; Bernardini, S. Differential Expression of miR-144* as a Novel Fecal-Based Diagnostic Marker for Colorectal Cancer. *J. Gastroenterol.* **2011**, *46*, 1391–1402.
190. Sun, N.; Zhang, L.; Zhang, C.; Yuan, Y. miR-144-3p Inhibits Cell Proliferation of Colorectal Cancer Cells by Targeting BCL6 via Inhibition of Wnt/β-Catenin Signaling. *Cell. Mol. Biol. Lett.* **2020**, *25*, 19.
191. Gao, Z.; Jiang, J.; Hou, L.; Zhang, B. Dysregulation of MiR-144-5p/RNF187 Axis Contributes To the Progression of Colorectal Cancer. *J Transl Int Med* **2022**, *10*, 65–75.
192. Ramzy, I.; Hasaballah, M.; Marzaban, R.; Shaker, O.; Soliman, Z.A. Evaluation of microRNAs-29a, 92a and 145 in Colorectal Carcinoma as Candidate Diagnostic Markers: An Egyptian Pilot Study. *Clin. Res. Hepatol. Gastroenterol.* **2015**, *39*, 508–515.
193. Xie, Y.; Zhang, Y.; Liu, X.; Cao, L.; Han, M.; Wang, C.; Chen, J.; Zhang, X. miR-151a-5p Promotes the Proliferation and Metastasis of Colorectal Carcinoma Cells by Targeting AGMAT. *Oncol. Rep.* **2023**, *49*, doi:10.3892/or.2023.8487.
194. Lv, Z.-C.; Fan, Y.-S.; Chen, H.-B.; Zhao, D.-W. Investigation of microRNA-155 as a Serum Diagnostic and Prognostic Biomarker for Colorectal Cancer. *Tumour Biol.* **2015**, *36*, 1619–1625.
195. de Groen, F.L.M.; Timmer, L.M.; Menezes, R.X.; Diosdado, B.; Hooijberg, E.; Meijer, G.A.; Steenbergen, R.D.M.; Carvalho, B. Oncogenic Role of miR-15a-3p in 13q Amplicon-Driven Colorectal Adenoma-to-Carcinoma Progression. *PLoS One* **2015**, *10*, e0132495.
196. Han, L.; Shi, W.-J.; Xie, Y.-B.; Zhang, Z.-G. Diagnostic Value of Four Serum Exosome microRNAs Panel for the Detection of Colorectal Cancer. *World J. Gastrointest. Oncol.* **2021**, *13*, 970–979.
197. Giráldez, M.D.; Lozano, J.J.; Ramírez, G.; Hijona, E.; Bujanda, L.; Castells, A.; Gironella, M. Circulating microRNAs as Biomarkers of Colorectal Cancer: Results from a Genome-Wide Profiling and Validation Study. *Clin. Gastroenterol. Hepatol.* **2013**, *11*, 681–688.e3.
198. Fu, F.; Jiang, W.; Zhou, L.; Chen, Z. Circulating Exosomal miR-17-5p and miR-92a-3p Predict Pathologic Stage and Grade of Colorectal Cancer. *Transl. Oncol.* **2018**, *11*, 221–232.
199. Liu, X.; Xu, T.; Hu, X.; Chen, X.; Zeng, K.; Sun, L.; Wang, S. Elevated Circulating miR-182 Acts as a Diagnostic Biomarker for Early Colorectal Cancer. *Cancer Manag. Res.* **2018**, *10*, 857–865.
200. Perilli, L.; Vicentini, C.; Agostini, M.; Pizzini, S.; Pizzi, M.; D'Angelo, E.; Bortoluzzi, S.; Mandruzzato, S.; Mammano, E.; Rugge, M.; et al. Circulating miR-182 Is a Biomarker of Colorectal Adenocarcinoma Progression. *Oncotarget* **2014**, *5*, 6611–6619.
201. Feng, J.; Wei, Q.; Yang, M.; Wang, X.; Liu, B.; Li, J. Development and Validation of a Novel miRNA Classifier as a Prognostic Signature for Stage II/III Colorectal Cancer. *Ann Transl Med* **2021**, *9*, 747.
202. Yau, T.O.; Wu, C.W.; Dong, Y.; Tang, C.-M.; Ng, S.S.M.; Chan, F.K.L.; Sung, J.J.Y.; Yu, J. microRNA-221 and microRNA-18a Identification in Stool as Potential Biomarkers for the Non-Invasive Diagnosis of Colorectal Carcinoma. *Br. J. Cancer* **2014**, *111*, 1765–1771.
203. Wikberg, M.L.; Myte, R.; Palmqvist, R.; van Guelpen, B.; Ljuslinder, I. Plasma miRNA Can Detect Colorectal Cancer, but How Early? *Cancer Med.* **2018**, *7*, 1697–1705.
204. Zhang, G.-J.; Zhou, T.; Liu, Z.-L.; Tian, H.-P.; Xia, S.-S. Plasma miR-200c and miR-18a as Potential Biomarkers for the Detection of Colorectal Carcinoma. *Mol Clin Oncol* **2013**, *1*, 379–384.
205. Basati, G.; Razavi, A.E.; Pakzad, I.; Malayeri, F.A. Circulating Levels of the miRNAs, miR-194, and miR-29b, as Clinically Useful Biomarkers for Colorectal Cancer. *Tumour Biol.* **2016**, *37*, 1781–1788.

206. Liu, Z.; Lu, T.; Wang, Y.; Jiao, D.; Li, Z.; Wang, L.; Liu, L.; Guo, C.; Zhao, Y.; Han, X. Establishment and Experimental Validation of an Immune miRNA Signature for Assessing Prognosis and Immune Landscape of Patients with Colorectal Cancer. *J. Cell. Mol. Med.* **2021**, *25*, 6874–6886.
207. Bilegsaikhan, E.; Liu, H.N.; Shen, X.Z.; Liu, T.T. Circulating miR-338-5p Is a Potential Diagnostic Biomarker in Colorectal Cancer. *J. Dig. Dis.* **2018**, *19*, 404–410.
208. Zheng, G.; Du, L.; Yang, X.; Zhang, X.; Wang, L.; Yang, Y.; Li, J.; Wang, C. Serum microRNA Panel as Biomarkers for Early Diagnosis of Colorectal Adenocarcinoma. *Br. J. Cancer* **2014**, *111*, 1985–1992.
209. Ahmed, F.E.; Jeffries, C.D.; Vos, P.W.; Flake, G.; Nuovo, G.J.; Sinar, D.R.; Naziri, W.; Marcuard, S.P. Diagnostic microRNA Markers for Screening Sporadic Human Colon Cancer and Active Ulcerative Colitis in Stool and Tissue. *Cancer Genomics Proteomics* **2009**, *6*, 281–295.
210. Xu, L.; Li, M.; Wang, M.; Yan, D.; Feng, G.; An, G. The Expression of microRNA-375 in Plasma and Tissue Is Matched in Human Colorectal Cancer. *BMC Cancer* **2014**, *14*, 714.
211. Chang, P.-Y.; Chen, C.-C.; Chang, Y.-S.; Tsai, W.-S.; You, J.-F.; Lin, G.-P.; Chen, T.-W.; Chen, J.-S.; Chan, E.-C. MicroRNA-223 and microRNA-92a in Stool and Plasma Samples Act as Complementary Biomarkers to Increase Colorectal Cancer Detection. *Oncotarget* **2016**, *7*, 10663–10675.
212. Yang, H.; Lin, J.; Jiang, J.; Ji, J.; Wang, C.; Zhang, J. miR-20b-5p Functions as Tumor Suppressor microRNA by Targeting cyclinD1 in Colon Cancer. *Cell Cycle* **2020**, *19*, 2939–2954.
213. Link, A.; Balaguer, F.; Shen, Y.; Nagasaka, T.; Lozano, J.J.; Boland, C.R.; Goel, A. Fecal MicroRNAs as Novel Biomarkers for Colon Cancer Screening. *Cancer Epidemiol. Biomarkers Prev.* **2010**, *19*, 1766–1774.
214. Du, M.; Liu, S.; Gu, D.; Wang, Q.; Zhu, L.; Kang, M.; Shi, D.; Chu, H.; Tong, N.; Chen, J.; et al. Clinical Potential Role of Circulating microRNAs in Early Diagnosis of Colorectal Cancer Patients. *Carcinogenesis* **2014**, *35*, 2723–2730.
215. Nassar, F.J.; Msheik, Z.S.; Itani, M.M.; Helou, R.E.; Hadla, R.; Kreidieh, F.; Bejjany, R.; Mukherji, D.; Shamseddine, A.; Nasr, R.R.; et al. Circulating miRNA as Biomarkers for Colorectal Cancer Diagnosis and Liver Metastasis. *Diagnostics (Basel)* **2021**, *11*, doi:10.3390/diagnostics11020341.
216. Zanutto, S.; Pizzamiglio, S.; Ghilotti, M.; Bertan, C.; Ravagnani, F.; Perrone, F.; Leo, E.; Pilotti, S.; Verderio, P.; Gariboldi, M.; et al. Circulating miR-378 in Plasma: A Reliable, Haemolysis-Independent Biomarker for Colorectal Cancer. *Br. J. Cancer* **2014**, *110*, 1001–1007.
217. Wang, Q.; Huang, Z.; Ni, S.; Xiao, X.; Xu, Q.; Wang, L.; Huang, D.; Tan, C.; Sheng, W.; Du, X. Plasma miR-601 and miR-760 Are Novel Biomarkers for the Early Detection of Colorectal Cancer. *PLoS One* **2012**, *7*, e44398.
218. Liu, G.-H.; Zhou, Z.-G.; Chen, R.; Wang, M.-J.; Zhou, B.; Li, Y.; Sun, X.-F. Serum miR-21 and miR-92a as Biomarkers in the Diagnosis and Prognosis of Colorectal Cancer. *Tumor Biology* **2013**, *34*, 2175–2181.
219. Basati, G.; Emami Razavi, A.; Abdi, S.; Mirzaei, A. Elevated Level of microRNA-21 in the Serum of Patients with Colorectal Cancer. *Med. Oncol.* **2014**, *31*, 205.
220. Wu, C.W.; Ng, S.S.M.; Dong, Y.J.; Ng, S.C.; Leung, W.W.; Lee, C.W.; Wong, Y.N.; Chan, F.K.L.; Yu, J.; Sung, J.J.Y. Detection of miR-92a and miR-21 in Stool Samples as Potential Screening Biomarkers for Colorectal Cancer and Polyps. *Gut* **2012**, *61*, 739–745.
221. Tsukamoto, M.; Iinuma, H.; Yagi, T.; Matsuda, K.; Hashiguchi, Y. Circulating Exosomal MicroRNA-21 as a Biomarker in Each Tumor Stage of Colorectal Cancer. *Oncology* **2017**, *92*, 360–370.
222. Fukada, M.; Matsuhashi, N.; Takahashi, T.; Sugito, N.; Heishima, K.; Yoshida, K.; Akao, Y. Postoperative Changes in Plasma miR21-5p as a Novel Biomarker for Colorectal Cancer Recurrence: A Prospective Study. *Cancer Sci.* **2021**, *112*, 4270–4280.
223. Wang, W.; Qu, A.; Liu, W.; Liu, Y.; Zheng, G.; Du, L.; Zhang, X.; Yang, Y.; Wang, C.; Chen, X. Circulating miR-210 as a Diagnostic and Prognostic Biomarker for Colorectal Cancer. *Eur. J. Cancer Care* **2017**, *26*, doi:10.1111/ecc.12448.
224. Yu, H.; Gao, G.; Jiang, L.; Guo, L.; Lin, M.; Jiao, X.; Jia, W.; Huang, J. Decreased Expression of miR-218 Is Associated with Poor Prognosis in Patients with Colorectal Cancer. *Int. J. Clin. Exp. Pathol.* **2013**, *6*, 2904–2911.
225. Pu, X.-X.; Huang, G.-L.; Guo, H.-Q.; Guo, C.-C.; Li, H.; Ye, S.; Ling, S.; Jiang, L.; Tian, Y.; Lin, T.-Y. Circulating miR-221 Directly Amplified from Plasma Is a Potential Diagnostic and Prognostic Marker of Colorectal Cancer and Is Correlated with p53 Expression. *J. Gastroenterol. Hepatol.* **2010**, *25*, 1674–1680.
226. Vychytilova-Faltejskova, P.; Radova, L.; Sachlova, M.; Kosarova, Z.; Slaba, K.; Fabian, P.; Grolich, T.; Prochazka, V.; Kala, Z.; Svoboda, M.; et al. Serum-Based microRNA Signatures in Early Diagnosis and Prognosis Prediction of Colon Cancer. *Carcinogenesis* **2016**, *37*, 941–950.
227. Fang, Z.; Tang, J.; Bai, Y.; Lin, H.; You, H.; Jin, H.; Lin, L.; You, P.; Li, J.; Dai, Z.; et al. Plasma Levels of microRNA-24, microRNA-320a, and microRNA-423-5p Are Potential Biomarkers for Colorectal Carcinoma. *J. Exp. Clin. Cancer Res.* **2015**, *34*, 86.
228. Liang, J.; Tang, J.; Shi, H.; Li, H.; Zhen, T.; Duan, J.; Kang, L.; Zhang, F.; Dong, Y.; Han, A. miR-27a-3p Targeting RXRα Promotes Colorectal Cancer Progression by Activating Wnt/β-Catenin Pathway. *Oncotarget* **2017**, *8*, 82991–83008.
229. Ostenfeld, M.S.; Jensen, S.G.; Jeppesen, D.K.; Christensen, L.-L.; Thorsen, S.B.; Stenvang, J.; Hvam, M.L.; Thomsen, A.; Mouritzen, P.; Rasmussen, M.H.; et al. miRNA Profiling of Circulating EpCAM(+) Extracellular Vesicles: Promising Biomarkers of Colorectal Cancer. *J Extracell Vesicles* **2016**, *5*, 31488.
230. Zhu, Y.; Xu, A.; Li, J.; Fu, J.; Wang, G.; Yang, Y.; Cui, L.; Sun, J. Fecal miR-29a and miR-224 as the Noninvasive Biomarkers for Colorectal Cancer. *Cancer Biomark.* **2016**, *16*, 259–264.

231. Huang, Z.; Huang, D.; Ni, S.; Peng, Z.; Sheng, W.; Du, X. Plasma microRNAs Are Promising Novel Biomarkers for Early Detection of Colorectal Cancer. *Int. J. Cancer* **2010**, *127*, 118–126.
232. Tadano, T.; Kakuta, Y.; Hamada, S.; Shimodaira, Y.; Kuroha, M.; Kawakami, Y.; Kimura, T.; Shiga, H.; Endo, K.; Masamune, A.; et al. MicroRNA-320 Family Is Downregulated in Colorectal Adenoma and Affects Tumor Proliferation by Targeting CDK6. *World J. Gastrointest. Oncol.* **2016**, *8*, 532–542.
233. Liu, X.; Xu, X.; Pan, B.; He, B.; Chen, X.; Zeng, K.; Xu, M.; Pan, Y.; Sun, H.; Xu, T.; et al. Circulating miR-1290 and miR-320d as Novel Diagnostic Biomarkers of Human Colorectal Cancer. *J. Cancer* **2019**, *10*, 43–50.
234. Cai, R.; Lu, Q.; Wang, D. Construction and Prognostic Analysis of miRNA-mRNA Regulatory Network in Liver Metastasis from Colorectal Cancer. *World J. Surg. Oncol.* **2021**, *19*, 7.
235. Song, H.; Ruan, C.; Xu, Y.; Xu, T.; Fan, R.; Jiang, T.; Cao, M.; Song, J. Survival Stratification for Colorectal Cancer via Multi-Omics Integration Using an Autoencoder-Based Model. *Exp. Biol. Med.* **2022**, *247*, 898–909.
236. Yu, J.; Jin, L.; Jiang, L.; Gao, L.; Zhou, J.; Hu, Y.; Li, W.; Zhi, Q.; Zhu, X. Serum miR-372 Is a Diagnostic and Prognostic Biomarker in Patients with Early Colorectal Cancer. *Anticancer Agents Med. Chem.* **2016**, *16*, 424–431.
237. Wang, L.; Song, X.; Yu, M.; Niu, L.; Zhao, Y.; Tang, Y.; Zheng, B.; Song, X.; Xie, L. Serum Exosomal miR-377-3p and miR-381-3p as Diagnostic Biomarkers in Colorectal Cancer. *Future Oncol.* **2022**, *18*, 793–805.
238. Wang, S.; Xiang, J.; Li, Z.; Lu, S.; Hu, J.; Gao, X.; Yu, L.; Wang, L.; Wang, J.; Wu, Y.; et al. A Plasma microRNA Panel for Early Detection of Colorectal Cancer. *Int. J. Cancer* **2015**, *136*, 152–161.
239. Tan, Y.; Lin, J.-J.; Yang, X.; Gou, D.-M.; Fu, L.; Li, F.-R.; Yu, X.-F. A Panel of Three Plasma microRNAs for Colorectal Cancer Diagnosis. *Cancer Epidemiol.* **2019**, *60*, 67–76.
240. Krawczyk, P.; Powrózek, T.; Olesiński, T.; Dmitruk, A.; Dziwota, J.; Kowalski, D.; Milanowski, J. Evaluation of miR-506 and miR-4316 Expression in Early and Non-Invasive Diagnosis of Colorectal Cancer. *Int. J. Colorectal Dis.* **2017**, *32*, 1057–1060.
241. Lan, S.-H.; Lin, S.-C.; Wang, W.-C.; Yang, Y.-C.; Lee, J.-C.; Lin, P.-W.; Chu, M.-L.; Lan, K.-Y.; Zuchini, R.; Liu, H.-S.; et al. Autophagy Upregulates miR-449a Expression to Suppress Progression of Colorectal Cancer. *Front. Oncol.* **2021**, *11*, doi:10.3389/fonc.2021.738144.
242. Zhang, Z.; Zhang, D.; Cui, Y.; Qiu, Y.; Miao, C.; Lu, X. Identification of microRNA-451a as a Novel Circulating Biomarker for Colorectal Cancer Diagnosis. *Biomed Res. Int.* **2020**, *2020*, 5236236.
243. Yang, I.-P.; Tsai, H.-L.; Hou, M.-F.; Chen, K.-C.; Tsai, P.-C.; Huang, S.-W.; Chou, W.-W.; Wang, J.-Y.; Juo, S.-H.H. MicroRNA-93 Inhibits Tumor Growth and Early Relapse of Human Colorectal Cancer by Affecting Genes Involved in the Cell Cycle. *Carcinogenesis* **2012**, *33*, 1522–1530.
244. Sun, Y.; Liu, Y.; Cogdell, D.; Calin, G.A.; Sun, B.; Kopetz, S.; Hamilton, S.R.; Zhang, W. Examining Plasma microRNA Markers for Colorectal Cancer at Different Stages. *Oncotarget* **2016**, *7*, 11434–11449.
245. Chen, Y.; Liu, H.; Ning, S.; Wei, C.; Li, J.; Wei, W.; Zhang, L. The High Ratio of the Plasma miR-96/miR-99b Correlated With Poor Prognosis in Patients With Metastatic Colorectal Cancer. *Front. Mol. Biosci.* **2022**, *8*, doi:10.3389/fmolb.2021.799060.
246. Hibino, Y.; Sakamoto, N.; Naito, Y.; Goto, K.; Oo, H.Z.; Sentani, K.; Hinoi, T.; Ohdan, H.; Oue, N.; Yasui, W. Significance of miR-148a in Colorectal Neoplasia: Downregulation of miR-148a Contributes to the Carcinogenesis and Cell Invasion of Colorectal Cancer. *Pathobiology* **2015**, *82*, 233–241.
